# On the development and validation of large language model-based classifiers for identifying social determinants of health

**DOI:** 10.1073/pnas.2320716121

**Published:** 2024-09-16

**Authors:** Rodney A. Gabriel, Onkar Litake, Sierra Simpson, Brittany N. Burton, Ruth S. Waterman, Alvaro A. Macias

**Affiliations:** ^a^Division of Perioperative Informatics, Department of Anesthesiology, University of California, San Diego, La Jolla, CA 92037; ^b^Department of Biomedical Informatics, University of California, San Diego Health, La Jolla, CA 92037; ^c^Department of Anesthesiology, University of California, Los Angeles, CA 90095

**Keywords:** social determinants of health, AI, large language models

## Abstract

The assessment of social determinants of health (SDoH) within healthcare systems is crucial for comprehensive patient care and addressing health disparities. Current challenges arise from the limited inclusion of structured SDoH information within electronic health record (EHR) systems, often due to the lack of standardized diagnosis codes. This study delves into the transformative potential of large language models (LLM) to overcome these challenges. LLM-based classifiers—using Bidirectional Encoder Representations from Transformers (BERT) and A Robustly Optimized BERT Pretraining Approach (RoBERTa)—were developed for SDoH concepts, including homelessness, food insecurity, and domestic violence, using synthetic training datasets generated by generative pre-trained transformers combined with authentic clinical notes. Models were then validated on separate datasets: Medical Information Mart for Intensive Care-III and our institutional EHR data. When training the model with a combination of synthetic and authentic notes, validation on our institutional dataset yielded an area under the receiver operating characteristics curve of 0.78 for detecting homelessness, 0.72 for detecting food insecurity, and 0.83 for detecting domestic violence. This study underscores the potential of LLMs in extracting SDoH information from clinical text. Automated detection of SDoH may be instrumental for healthcare providers in identifying at-risk patients, guiding targeted interventions, and contributing to population health initiatives aimed at mitigating disparities.

## Significance

Detecting social determinants of health (SDoH) concepts within unstructured clinical notes poses challenges, exacerbated by sparse availability of annotated datasets. Our approach involved generating synthetic datasets by leveraging generative pre-trained transformers to introduce SDoH concepts into training data. Subsequent validation of the models with two diverse clinical datasets, including MIMIC-III and our institutional electronic health records, demonstrated the promising role of large language models for extracting crucial SDoH information from unstructured data. Furthermore, it highlights the potential to enhance and provide automated detection tools that can aid healthcare providers in the assessment of SDoH.

The Center for Medicare and Medicaid Services has finalized coding and payment for social determinants of health (SDoH) risk assessments (1). SDoH are complex conditions of the environment—economic stability, access to quality education, neighborhood and built environment, social and community context, and health care access and quality—that affect risk of disease, health outcomes, and quality of life (2). Outside general demographic information, such as race and ethnicity, payer status, and primary language, current electronic health record (EHR) systems rarely include “structured” SDoH information, which can lead to a lack of structured and consistent diagnosis codes. SDoH are often imbedded in unstructured clinical notes and, thus, are consequently time-consuming and cumbersome to extract from the EHR (3).

Advances in AI tools for natural language processing (NLP), specifically large language models (LLM), coupled with the widespread adoption of EHRs may help to address persistent issues in medicine, particularly health disparities. LLMs are powerful algorithms designed with millions to billions of parameters, trained on extremely large text corpus and have been applied to solve various healthcare-related problems (4, 5). One of its use cases would be to process clinical documents and classify a patient’s current health and/or social status. Understanding unstructured clinical data, such as physician-generated text documents, is a powerful approach to creating meaningful use of EHR data—in this, identifying and assessing SDoH. Leveraging LLMs to identify SDoH from clinical notes could assist healthcare systems in improving assessments of SDoH for their patients and better understand patterns in social needs that drive healthcare for the populations.

Previous studies have demonstrated the usefulness of NLP in extracting SDoH information using various NLP approaches, including lexicon creation and supervised/unsupervised machine learning methods (6–8). More work is needed to investigate NLP tools that readily extract SDoH beyond variables that are likely included in the medical record as structured data. While it is important to consider structured demographic data that can be readily extracted from the EHR, these data are often a proxy for the social and cultural environment. In this study, we describe an approach to 1) easily develop training sets for supervised training of LLM-based classifiers while minimizing the need for lexicon creation or manual labeling of patient notes; 2) leverage LLMs to customize models to identify SDoH; and 3) validation of these models. The first objective is especially important when there is a lack of large note datasets with prelabeled SDoH data. We aimed to demonstrate the ability of LLM classifiers to generalize across publicly available and institutional datasets. This has the potential to allow healthcare providers to assess for SDoH, which has downstream potential for improving health equity.

## Results

### Model Training.

**Fig. 1.**
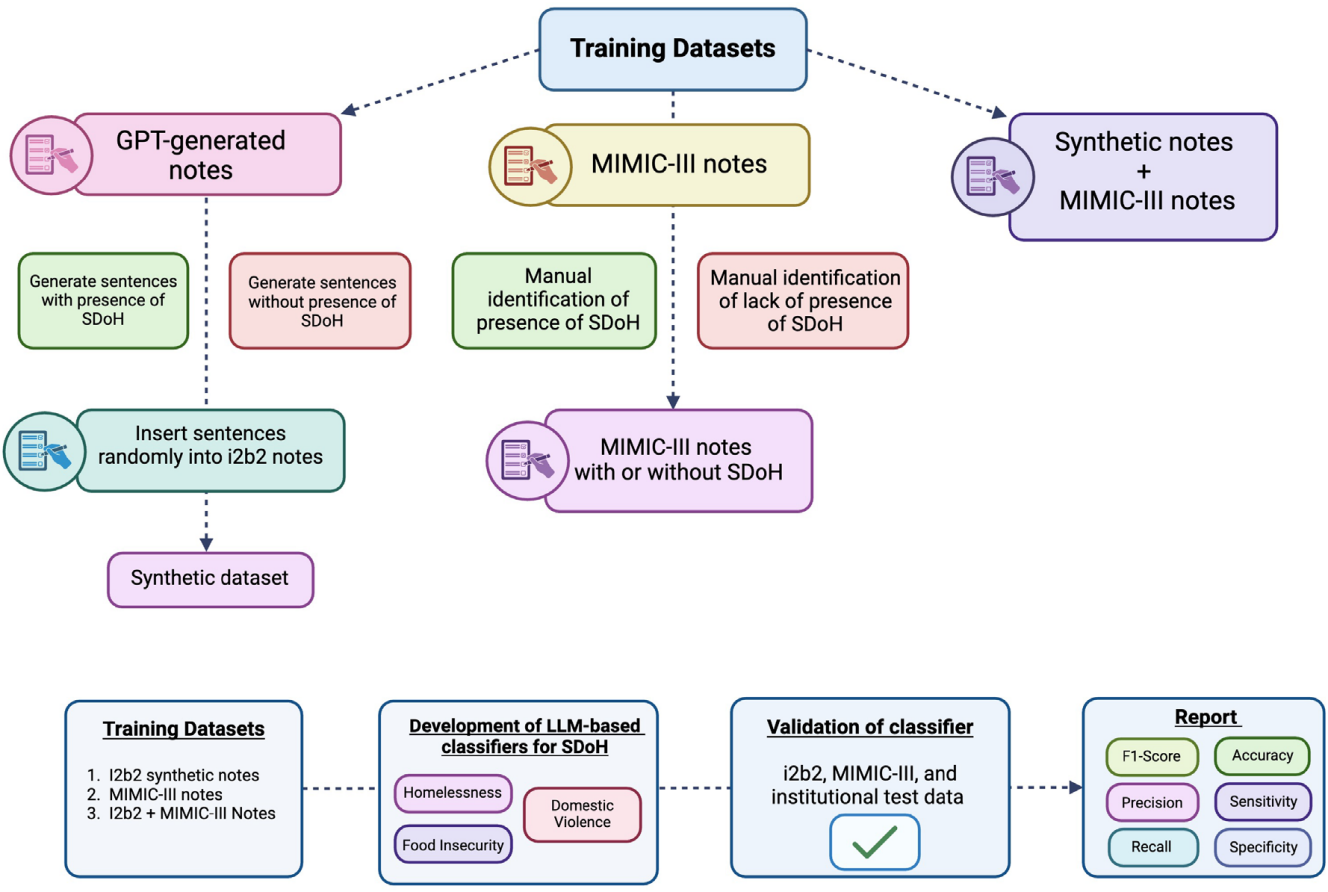
Illustration of study methods. LLM-based classifiers for SDoH were developed on three different types of training sets: 1) Synthetic dataset, in which GPT-generated text phrases for SDoH were inserted into i2b2 clinical notes; 2) Authentic clinical notes from MIMIC-III; and 3) Combination of synthetic notes and MIMIC-III notes. Subsequently, these models were validated on test sets from i2b2, MIMIC-III, and institutional data for performance evaluation. Abbreviations: SDoH, Social Determinants of Health; EHR, electronic health record; GPT, generative pre-trained transformers; LLM, large language model, MIMIC-III, Medical Information Mart for Intensive Care-III.

Our objective was to develop LLM-based classifier models capable of detecting SDoH within a patient’s medical notes. We compared the performance of these models based on different types of training datasets: 1) synthetically generated notes using generative pre-trained transformer (GPT) 3.5 turbo (synthetic-i2b2-notes), 2) authentic notes from the Medical Information Mart for Intensive Care (MIMIC-III) dataset (mimic-notes), and 3) a combination of GPT-generated synthetic notes with authentic notes from MIMIC-III (synthetic+mimic-notes). These models were then validated on separate test sets from i2b2, authentic MIMIC-III notes, and finally, authentic notes from our institution’s EHR. Each LLM was trained to classify three types of SDoH: 1) homelessness, 2) food insecurity, and (3) domestic violence (Fig. 1). The composition of each dataset are described in Table 1.

**Table 1.** Proportion of positive and negative SDoH labels and sample size for the training and validation datasets

	Homelessness		Food Insecurity		Domestic Violence	
Datasets	Total sample size, n	Patients with SDoH, n (%)	Total sample size, n	Patients with SDoH, n (%)	Total sample size, n	Patients with SDoH, n (%)
Training dataset						
synthetic notes only	592	70 (11.8%)	592	70 (11.8%)	592	70 (11.8%)
mimic-notes	100	50 (0.5%)	40	10 (0.5%)	100	50 (0.5%)
synthetic + mimic-notes	692	120 (17.3%)	632	80 (12.7%)	692	120 (17.3%)
Validation dataset						
i2b2	198	30 (15.2%)	198	30 (15.2%)	198	30 (15.2%)
MIMIC-III	5,114	2164 (42.3%)	150	9 (6.0%)	2,000	160 (8.0%)
Institutional EHR data	2,000	50 (2.5%)	2,000	50 (2.5%)	2,000	50 (2.5%)

Abbreviations: EHR, electronic health record; MIMIC-III, Medical Information Mart for Intensive Care-III; SDoH, social determinants of health.

There are three types of training data used: 1) synthetic notes only—defined by synthetic sentences with positive or negative labels for SDoH created by generative AI and injected into notes from i2b2; 2) authentic notes from MIMIC-III data where labeling of SDoH was performed by manual clinician review; and 3) combination of synthetic notes and authentic MIMIC-III notes. There are three validation datasets used: 1) a test set from the synthetic dataset from i2b2; 2) a test set from the authentic MIMIC-III notes; and 3) notes from our institution electronic health record data. Labeling of SDoH was performed by manual clinician review.

### Validation of Model for Detecting Homelessness.

We trained three LLM-based classifiers based on the training data used to identify homelessness from clinical notes. We report results from the A Robustly Optimized BERT Pretraining Approach (RoBERTa) model and, additionally, provide performance of the Bidirectional Encoder Representations from Transformers (BERT) models in *SI Appendix*, Fig. S1 [area under the receiver operating characteristics curve (AUC)], *SI Appendix*, Fig S2 (precision–recall curves) and *SI Appendix*, Table S1 (F1-score, precision, recall, accuracy, sensitivity, and specificity). The classifier had the highest AUC for identifying homelessness from the institution EHR dataset when trained on the synthetic+mimic-notes training dataset (area under the receiver operating characteristics curve (AUC) = 0.78 versus 0.64 with synthetic-notes and 0.69 with mimic-notes).

**Fig. 2.**
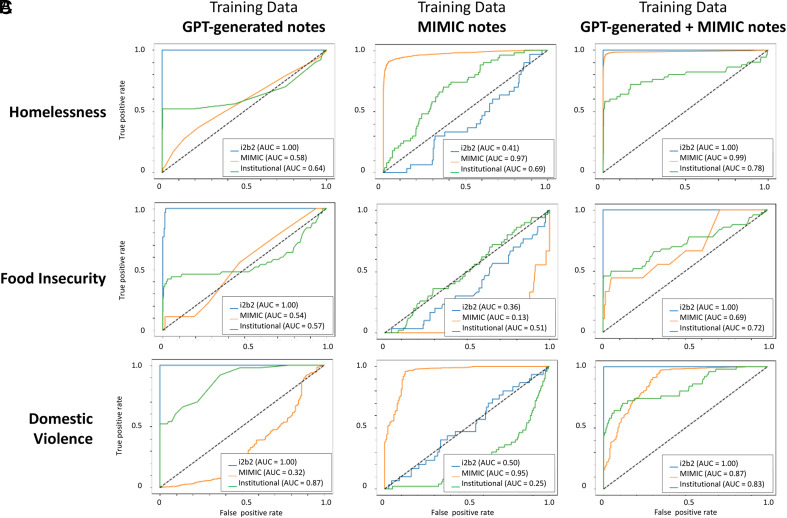
Area under the receiver operating characteristics curve for the large language model-based classifiers using RoBERTa for detecting homelessness, food insecurity, or domestic violence from clinical notes. Each column corresponds to the training set used for the classifier: 1) Synthetic notes only, 2) Authentic clinical notes from MIMIC-III, and 3) Combination of synthetic notes and MIMIC-III. Within each plot, the AUC is illustrated for when the classifier was validated on three different test sets from i2b2 (synthetic notes), MIMIC-III, and institutional electronic health record notes. Performance is illustrated based on (*A*) homelessness, (*B*) food insecurity, and (*C*) domestic violence. Abbreviations: AUC, area under the receiver operating characteristics curve; RoBERTa, a Robustly optimized BERT approach; MIMIC, Medical Information Mart for Intensive Care.

**Fig. 3.**
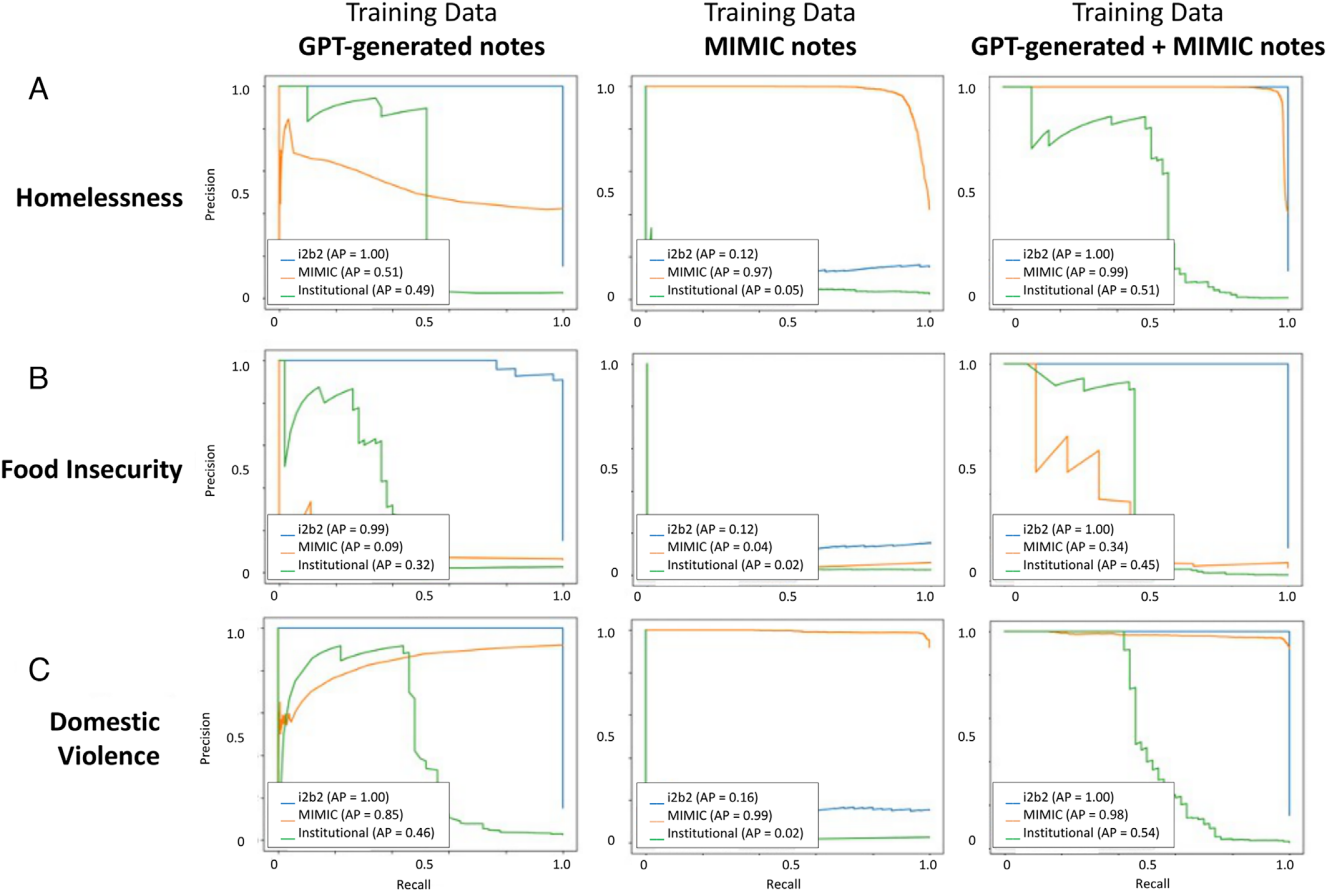
Precision–recall curves for the large language model-based classifiers using RoBERTa for detecting homelessness, food insecurity, or domestic violence from clinical notes. Each column corresponds to the training set used for the classifier: 1) Synthetic notes only, 2) Authentic clinical notes from MIMIC-III, and 3) Combination of synthetic notes and MIMIC-III. Within each plot, the precision–recall curve is illustrated for when the classifier was validated on three different test sets from i2b2 (synthetic notes), MIMIC-III, and institutional electronic health record notes. Performance is illustrated based on (*A*) homelessness, (*B*) food insecurity, and (*C*) domestic violence. Abbreviations: RoBERTa, a Robustly optimized BERT approach; GPT, generative pre-trained transformers; MIMIC, Medical Information Mart for Intensive Care.

**Fig. 4.**
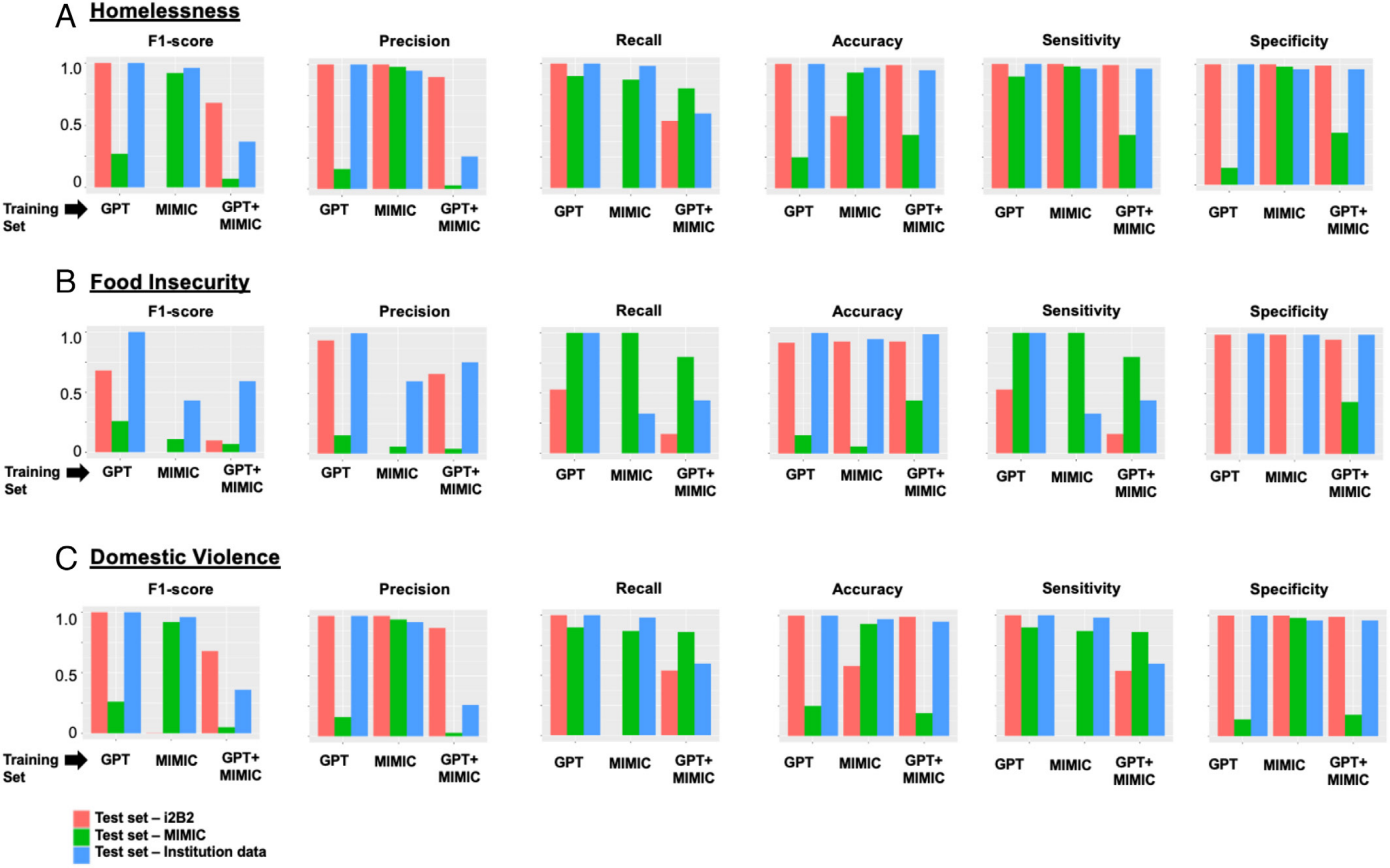
Performance metrics (F1-score, precision, recall, accuracy, sensitivity, and specificity) of large language model-based (RoBERTa) classifiers for identifying homelessness, food insecurity, or domestic violence from clinical notes. Within each plot, there are three sets of three bar plots. Each set of three represents performance on various test sets based on the training set used: 1) GPT = training set from synthetic notes only, 2) MIMIC = training set from authentic notes from MIMIC-III, and 3) GPT+MIMIC = combination of GPT and MIMIC. Within each set, the performance of the classifier is represented based on the test set: 1) synthetic notes only from i2b2 (red), 2) authentic clinical notes from MIMIC-III (green), and 3) institutional electronic health record data. Performance is illustrated based on (*A*) homelessness, (*B*) food insecurity, and (*C*) domestic violence. Abbreviations: GPT, generative pre-trained transformers; MIMIC, Medical Information Mart for Intensive Care; RoBERTa, A Robustly Optimized BERT Pretraining Approach.

When trained on synthetic-notes and tested on the test sets for i2b2, MIMIC-III, and institutional EHR data, the AUCs were 1.00, 0.58, and 0.64, respectively (Fig. 2*A*). The precision–recall curves are provided in Fig. 3. The F1-scores were 1.00, 0.003, and 0.68, respectively (Fig. 4*A*). When trained on mimic-notes and tested on the test sets for i2b2, MIMIC-III, and institutional EHR data, the AUCs were 0.41, 0.97, and 0.69, respectively, and the F1-scores were 0.27, 0.92, and 0.07, respectively. When trained on synthetic+mimic-notes and tested on the test sets for i2b2, MIMIC-III, and institutional EHR data, the AUCs were 1.00, 0.99, and 0.78, respectively, and the F1-scores were 1.00, 0.96, and 0.37, respectively.

### Validation of Model for Detecting Food Insecurity.

Similarly, we trained three LLM-based classifiers based on the training data used to identify food insecurity from clinical notes. The classifier had the highest AUC for identifying food insecurity from the institution EHR dataset when trained on the synthetic+mimic-notes training dataset (AUC = 0.72 versus 0.57 with synthetic-notes and 0.51 with mimic-notes).

When trained on synthetic-notes and tested on the test sets for i2b2, MIMIC-III, and institutional EHR data, the AUCs were 1.00, 0.54, and 0.57, respectively (Fig. 2*B*). The precision–recall curves are provided in Fig. 3. The F1-scores were 0.68, 0, and 0.1, respectively (Fig. 4*B*). When trained on mimic-notes and tested on the test sets for i2b2, MIMIC-III, and institutional EHR data, the AUCs were 0.38, 0.13, and 0.51, respectively, and the F1-scores were 0.26, 0.11, and 0.07, respectively. When trained on synthetic+mimic-notes and tested on the test sets for i2b2, MIMIC-III, and institutional EHR data, the AUCs were 1.00, 0.69, and 0.72, respectively, and the F1-scores were 1.00, 0.43, and 0.59, respectively.

### Validation of Model for Detecting Domestic Violence.

Finally, we trained three LLM-based classifiers based on the training data used to identify domestic violence from clinical notes. The classifier had the highest AUC for identifying food insecurity from the institution EHR dataset when trained on the synthetic-notes training dataset (AUC = 0.87 versus 0.25 with mimic-notes and 0.83 with synthetic+mimic-notes).

When trained on synthetic-notes and tested on the test sets for i2b2, MIMIC-III, and institutional EHR data, the AUCs were 1.00, 0.32, and 0.87, respectively (Fig. 2*C*). The precision–recall curves are provided in Fig. 3. The F1-scores were 1.00, 0.003, and 0.68, respectively (Fig. 4*C*). When trained on mimic-notes and tested on the test sets for i2b2, MIMIC-III, and institutional EHR data, the AUCs were 0.50, 0.95, and 0.25, respectively, and the F1-scores were 0.26, 0.92, and 0.05, respectively. When trained on synthetic+mimic-notes and tested on the test sets for i2b2, MIMIC-III, and institutional EHR data, the AUCs were 1.00, 0.87, and 0.83, respectively, and the F1-scores were 1.00, 0.96, and 0.36, respectively.

## Discussion

In this study, we described a method for training LLM-based classifiers for identifying SDoH using synthetic datasets generated from GPT from clinical notes. This is especially important given that annotated datasets for SDoH are currently lacking and thus would otherwise require manual chart review. We subsequently compared the performance of LLM-based SDoH classifiers using three different training sets: synthetic notes only, authentic notes from MIMIC-III, and a combination of synthetic notes and MIMIC-III notes. Performance varied based on the SDoH and the training dataset used for the model. For example, when trained purely on synthetic data (i2b2), classification of SDoH performed well on the institutional EHR data, but not the MIMIC-III data. When the model was trained on MIMIC-III data, the models did not perform well on the synthetic test set nor the institution EHR dataset. Interestingly, when using the combination of synthetic notes and MIMIC-III notes as training data, the model outperformed for identifying homelessness and food insecurity. Performance was similar for synthetic notes-only versus combination of synthetic notes and MIMIC-III as training data for identifying domestic violence. The results suggest that supplementing training data with synthetic data may optimize predictive performance for identifying SDoH from the unstructured data present in clinical notes. Degradation in performance that was based on the training set and validation set suggests that the generalization of these models would potentially require a combination of authentic and synthetic notes, rather than just one or the other.

SDoH are the nonclinical factors—housing, transportation, employment, violence, food insecurity, physical activity opportunity, air quality, and clean water—that are associated with health outcomes (9–11). These are the conditions in which people are born, grow, work, live, and age, as well as the systems, beyond the individual, that shape health risk (2, 9, 12). The present study outlined a technique for generating a synthetic dataset using GPT to train models for recognizing three SDoH—homelessness, food insecurity, and domestic violence—from clinical notes. This is particularly crucial as annotated public datasets for SDoH are not widely available. Creation of such datasets would require manual chart reviews that may be biased or incomplete. Adequate discrimination of homelessness, food insecurity, and domestic violence by this models is crucial as SDoH are associated with several negative health outcomes including– but not limited to - postoperative complications, development of medical comorbidities, and an increase in healthcare utilization (13–15). Only 10 to 20% of modifiable risk factors of health outcomes are attributable to direct medical care, while roughly 80 to 90% of health outcomes, are attributed to SDoH (16). Undoubtedly, improvements in health disparities and population health will require health policy changes that address SDoH (17). Automating the identification of SDoH concepts from patient records is vital, especially since many of these concepts lack structural and consistent representation across EHRs and institutions (18).

The current study builds on the foundation of previous research exploring NLP techniques to extract SDoH concepts from clinical text, including manual lexicon curation, semiautomated lexicon creation, rule-based methods, and supervised learning techniques (6, 19–21), Mehta et al. applied rule-based NLP tools to extract SDoH details from unstructured data of diabetic patients and found that SDoH were more significant to clinical outcomes that were originally hypothesized from structured data extracted from the EHR (19). Other supervised learning approaches have also been applied, which included deep learning approaches (21–25). More recently, LLMs, such as BERT, have also been investigated (20, 24). Three deep learning models in identifying SDoH from manually annotated clinical notes from the MIMIC database were described (20). Convolutional neural networks, long short-term memory, and BERT were tested for the detection of SDoH concepts. In this instance, BERT outperformed the other models in most metrics—specifically in the occupational category—but underperformed in the non-SDoH category. BERT also outperformed the other deep learning models in distinguishing social vs nonsocial sentences, likely due to better recognition of context.

In contrast to the arduous manual approach of data curation, the present study described an expedited approach to creating a synthetic clinical note dataset for training models. This involved querying GPT to compose various sentence-phrases that were either a positive or a negative description of a SDoH concept of interest. These sentences were then injected into clinical notes and subsequently used as a training dataset. LLM-based classifiers, BERT and RoBERTa, were then developed and validated on two separate EHR-based datasets, MIMIC-III and our institutional EHR. RoBERTa marginally outperformed BERT across most SDoH concepts; however, comparing the performance of BERT and RoBERTa requires the consideration of various factors, including architecture and training objectives. Both models share a transformer architecture but diverge in certain implementation specifics, including training objectives and hyperparameter tuning (26, 27). The pretraining approach, quality of the training data, and architectural nuances may contribute to the models’ ability to generalize to downstream tasks.

The specific task of SDoH detection, particularly in the context of synthetically generated sentences, may influence the relative performance of these models. Furthermore, the selection of appropriate evaluation metrics is pivotal, as certain models may exhibit strengths in specific metrics while performing comparably in others. BERT’s bidirectional attention mechanism, which considers both preceding and following words during pretraining, may enhance its ability to capture intricate contextual dependencies, a particularly valuable quality in tasks like the detection of SDoH where the meaning of a sentence relies heavily on the surrounding context. RoBERTa, while sharing the transformer architecture, may exhibit variations in its training approach that influence its sensitivity to specific contextual nuances. Moreover, the inherent complexity of SDoH-related information, often manifesting in subtle linguistic expressions, necessitates a model’s adeptness at grasping nuanced language patterns (28). The capacity of BERT and RoBERTa to capture these subtleties may differ, affecting their performance in the task of identifying SDoH from clinical text.

The ability to accurately identify SDoH from clinical notes has important implications for healthcare systems. Socioeconomic needs are closely tied to health outcomes but often go undocumented in structured fields. Uncovering SDoH information from clinical narratives can help providers better understand social barriers and tailor interventions. NLP for SDoH enables health-policy and population-health analysts to identify trends in high-risk groups encourage evidenced-based policy change and interventions. Furthermore, incorporating SDoH information into machine learning may improve model prediction and integration of SDoH into clinical workflows could streamline social risk screening and referrals (3, 15, 19, 21). The use of NLP for the identification of SDoH in unstructured clinical notes will help to identify socioeconomic factors, beyond race and ethnicity, associated with health risk and outcomes, improve operational efficiency by negating the need for manual chart review, and reduce the need for providers to identify nuanced SDoH language that may otherwise go unnoticed due to training or social differences (6, 29).

Caution is appropriate when considering the results of this study in the context of clinical care. The models were trained on an artificial dataset, which may not fully represent real-world clinical language. Regardless, the models were validated on a larger corpus of authentic notes with expert annotations. However, further validation is required in other environments given the potential lack of consistency of SDoH documentation across institutions. Rural and urban institutions may have different distributions of SDoH concepts (30, 31). To simplify our study, we identified only three SDoH that may be broadly grouped into economic stability (e.g., homelessness and food insecurity) and social and community context (e.g., domestic violence) (2). According to *Healthy People 2030*, there are at least three other broad domains of SDoH—education access and quality, health care access and quality, and neighborhood and built environment—all of which influence health outcomes (2). Given the significant contribution of SDoH on health outcomes, more comprehensive work is needed to identify all five domains of SDoH from diverse populations.

Furthermore, there are several limitations with the MIMIC dataset being utilized as an external validation set. As described in the methods, labeling of SDoH characteristics were contingent on the initial screening of these notes, which involved filtering candidate notes with predefined expressions related to a specific SDoH characteristic. These expressions may not have comprehensively captured all potential patients with domestic violence, food insecurity, or homelessness. For example, filtering of potential patients with domestic violence was initially screened by the expression of “violence” or “abuse.” However, it may not have captured patients that indeed had domestic violence but with the text worded without using these predefined expressions. Regardless, the MIMIC dataset served as only one validation set to demonstrate a proof-of-concept. Our institutional external validation set, on the other hand, consisted of notes from patients that were more accurately identified as having a SDoH history based on routine preoperative clinical interviews. The performance of the models was adequate for both validation sets, which highlights the potential use of these language model classifiers for identifying SDoH from clinical notes.

In conclusion, our study demonstrated the potential of LLM-based classifiers trained on synthetic datasets for identifying the presence of crucial SDoH information embedded within clinical text. The automated detection of SDoH not only may provide invaluable insights into socioeconomic needs but also serves as a pivotal tool for downstream tasks aimed to mitigate disparities and enhance health outcomes. The integration of this technology into healthcare systems can notably augment community health needs assessments, enabling institutions to refine programs and interventions over time. The findings of this study underscore the transformative impact that leveraging embedded EHR data can have on advancing public health initiatives and improving medical practices.

## Materials and Methods

This retrospective study was approved by the Human Research Protections Program at the University of California, San Diego for the collection of data from the electronic medical record system. The informed consent requirement was waived. Institutional data were obtained from surgical patients from April 2022 to March 2023. Our objective was to develop LLM-based classifier models capable of detecting SDoH within a patient’s medical notes. We compared the performance of these models based on different types of training datasets: 1) synthetically generated notes using GPT, 2) authentic notes from the MIMIC-III dataset, and 3) a combination of GPT-generated synthetic notes with authentic notes from MIMIC-III. These models were then validated on separate test sets from i2b2, authentic MIMIC-III notes, and finally, authentic notes from our institution’s electronic medical record. Each LLM was trained to classify three types of SDoH: 1) homelessness, 2) food insecurity, and 3) domestic violence.

### Training Datasets for LLM-Classifier.

To train the classifier, we created three different training datasets as described above. The first training set (synthetically generated notes using GPT) were synthetically designed notes that contain notes that were either positive or negative for each SDoH. This was executed by injecting sentences related to (or negation statements) SDoH to existing clinical notes from the publicly available n2c2 clinical note dataset (2014 Deidentification and Heart Disease) from the i2b2 (32). The i2b2 platform is an open-source clinical data warehousing and analytics tool designed to facilitate the sharing, integration, standardization, and analysis of diverse healthcare and research data sources. The i2b2 dataset comprised 790 deidentified notes, each containing patient information from several participants, including their current medical condition, history of present illness, and other pertinent details. Text phrases were “artificially” generated using GPT related to various SDoH, producing 100 sentences for each SDoH topic. The specific prompts used for text generation can be found in supplementary information (*SI Appendix*, Table S2). For instance, 100 sentences were generated pertaining to the “Homelessness” (*SI Appendix*, Table S3). An example sentence generated is as follows: “The patient presented with multiple health issues, including respiratory infections and malnutrition, which are commonly observed among individuals lacking stable housing.” These 100 sentences were then randomly incorporated into selected 100 notes from the i2b2 dataset at a rate of one unique set of sentences per note. These selected notes were assigned a label of “1,” meaning that the patient note had the Homelessness SDoH. Conversely, 100 negation statements sentences related to Homelessness were generated and subsequently inserted randomly into a separate set of 40 random notes at a rate of one unique set of sentences per note. These synthetically generated negation sentences are provided in *SI Appendix*, Table S4. An example negation statement was “The absence of homelessness issues allows the patient to access consistent healthcare services.” All notes without the positive statements were labeled “0,” which meant the patient did not have homelessness (all notes that did not receive an artificial sentence and all notes that received a negation statement). Manual review of the notes was also performed to ensure the patient corresponding to that note was not homeless in reality. This same process was replicated separately for the “food insecurity” and “domestic violence” SDoH groups. Positive and negation GPT-generated statements for food insecurity are listed in *SI Appendix*, Tables S5 and S6, respectively. Likewise, positive and negation statements for domestic violence are listed in *SI Appendix*, Tables S7 and S8, respectively. The final dataset included a homelessness, food insecurity, and domestic violence dataset (Table 1).

The second training dataset was authentic notes from the MIMIC-III dataset. We gathered notes from patients who had an SDoH described in their notes. To do this, we first identified potential notes that may have SDoH information by filtering via regular expression. For homelessness, we searched for all notes containing the expressions “homeless” and/or “housing” (which yielded 3,261 notes). These notes along with randomly chosen control notes were manually screened by authors to determine the presence or absence of homelessness. This was performed similarly for food insecurity, in which we searched for all notes containing the expression “lack of food” and/or “access to food” (which yielded 19 notes). For domestic violence, we searched for all discharge summary notes containing the expression violence and/or abuse (which yielded 7,190 notes). We had separate note datasets for homelessness, food insecurity, and domestic violence. The dataset also contained notes that did not have an SDoH. These datasets were then split into a 75%/25% training/test set. The training sets were used to train the LLM classifier and the test sets were used for validation of the models. The third training dataset combined both the GPT-created synthetic notes (i2b2) and some notes from the MIMIC-III training set (Table 1).

### Development of LLM-Based Classifier for SDoH.

Two LLMs were used: BERT (26) and RoBERTa (27). BERT is a pretrained language model developed by Google. BERT’s pretraining on a massive corpus of text enables it to capture complex language patterns and semantics. Developed by Facebook, RoBERTa was designed to improve upon BERT’s pretraining methodology, optimizing it with larger batch sizes and more training data (27). The LLM-based classifiers were trained on each of the training sets described above and then validated on the three different test sets: 1) test set from of the GPT-generated notes (i2b2), 2) authentic notes from the test set of MIMIC-III, and 3) authentic notes from patients from our institution’s electronic medical record (described below). Model hyperparameter optimization was done for learning rate, maximum sequence length, optimizer, adam epsilon, and train batch size. The model was fine-tuned for 10 epochs and the best-performing model was used for evaluation. The models took approximately 20 min to fine-tune. A 75–25 split for each dataset was adopted. The evaluation of the model was conducted on 25% of the dataset. To accommodate the maximum length limitations imposed by most language models, the notes were trimmed to approximately 512 tokens. We used NVIDIA Tesla V100 GPU to train our model. We used “simpletransformers” library, which is a wrapper around Hugging Face transformer’s library.

### Validation Datasets.

Following the development of the LLM-based classifiers for SDoH on the i2b2 dataset, we internally validated the models first on the test set portion of the i2b2 dataset and then externally validated the models using the following separate dataset groups: 1) discharge summaries from the MIMIC-III dataset; and 2) authentic clinical notes from our institution’s EHR system.

The MIMIC dataset comprises anonymized health-related data pertaining to over 40,000 patients admitted to the Beth Israel Deaconess Medical Center’s critical care units during the period from 2001 to 2012 (13). Our analysis highlighted the text sections under specific keywords, namely—history of present illness, brief hospital course, assessment and plan, and social history, as being particularly significant. Subsequently, data were extracted from these sections for each note and the remaining text was removed.

The second external validation dataset was authentic history and physical notes from patients within our institution’s EHR. At our institution, we regularly maintained a registry for SDoH for quality improvement purposes, in which patients identified via interview during surgical evaluation at our anesthesia preoperative care clinic were stored. We leveraged this existing dataset to identify patients with SDoH. For controls, we extracted random patients from the same period who were evaluated at our anesthesia preoperative care clinic who were not identified as having any of the SDoH. At this point, authors reviewed each of the charts to confirm positive and negative labels. Labeling of SDoH for the notes (both MIMIC-III and institution EHR notes) were done manually by three clinicians—two clinicians reviewed all notes and marked presence or lack of the SDoH. In the event that there was disagreement, the third clinician reviewed those notes and broke the tie. Three datasets were prepared for each SDoH (homelessness, food insecurity, and domestic violence). Each dataset consisted of 2,000 history and physical notes each from a unique patient. Fifty patients with the known presence of a SDoH concept were included with the remaining 1,950 without the presence of a known SDoH concept. Table 1 lists the composition of each dataset.

The LLM-based classifiers were applied to both external validation datasets (authentic MIMIC-III and institution EHR notes). Performance was compared based on the how the classifier was trained (i.e., the training set used). For each SDoH, we assessed the performance of each language model (BERT and RoBERTa) and reported the F1-score, AUC, precision, recall, accuracy, sensitivity, and specificity. The probability threshold set for calculating F1-score, precision, recall, accuracy, sensitivity, and specificity was 0.5. An illustration of the overall methodology is provided in Fig. 1. Python 3.10.12 was used for all statistical analysis.

## Supplementary Material

Appendix 01 (PDF)

## Data Availability

The code for the LLM-based classifier for SDoH is provided in the following link: https://github.com/UCSDGabrielLab/SDoHLLM (33). The training dataset used was from i2b2 and may be requested via the following link: https://portal.dbmi.hms.harvard.edu/projects/n2c2-nlp/ (34). One validation dataset, the MIMIC-III clinical database, is available at the following link: https://physionet.org/content/mimiciii/1.4/ (35).
