# Supplementary material for: On the development and validation of large language model-based classifiers for identifying social determinants of health
Source: Proc Natl Acad Sci U S A. 2024 Sep 16;121(39):e2320716121. doi: 10.1073/pnas.2320716121 (PMC11441499; doi:10.1073/pnas.2320716121)
Supplement: Supplementary file 1 — Appendix 01 (PDF) [file pnas.2320716121.sapp.pdf]

PNAS

Supporting Information for

On the Development and Validation of Large Language Model-Based Classifiers for Identifying Social Determinants of Health

Rodney A. Gabriel, MD, MAS, Onkar Litake, BS, Sierra Simpson, PhD, Brittany N. Burton, MD, MAS, MHS, Ruth S. Waterman, MD, MS, Alvaro A. Macias, MD

Corresponding Author: Rodney Gabriel
Email: ragabriel@health.ucsd.edu

This PDF file includes:

Supporting text
Figures S1 to S2
Tables S1 to S8

BERT

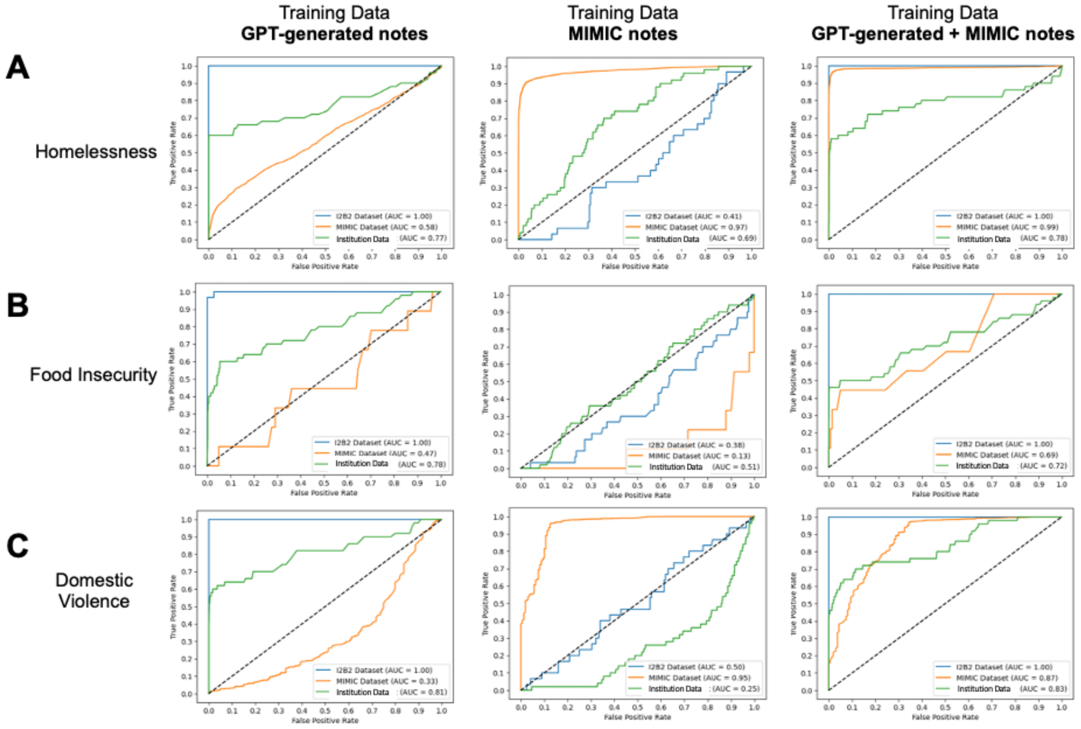

Fig. S1. Area under the receiver operating characteristics curve for the large language model-based classifiers using BERT for detecting homelessness, food insecurity, or domestic violence from clinical notes. Each column corresponds to the training set used for the classifer: (1) synthetic notes only, (2) authentic clinical notes from MIMIC-III, and (3) combination of synthetic notes and MIMIC-III. Within each plot, the AUC is illustrated for when the classifier was validated on three different test sets from i2b2 (synthetic notes), MIMIC-III, and institutional electronic health record notes. Performance is illustrated based on A) homelessness, B) food insecurity, and C) domestic violence. Abbreviations: AUC, area under the receiver operating characteristics curve; MIMIC, Medical Information Mart for Intensive Care.

BERT

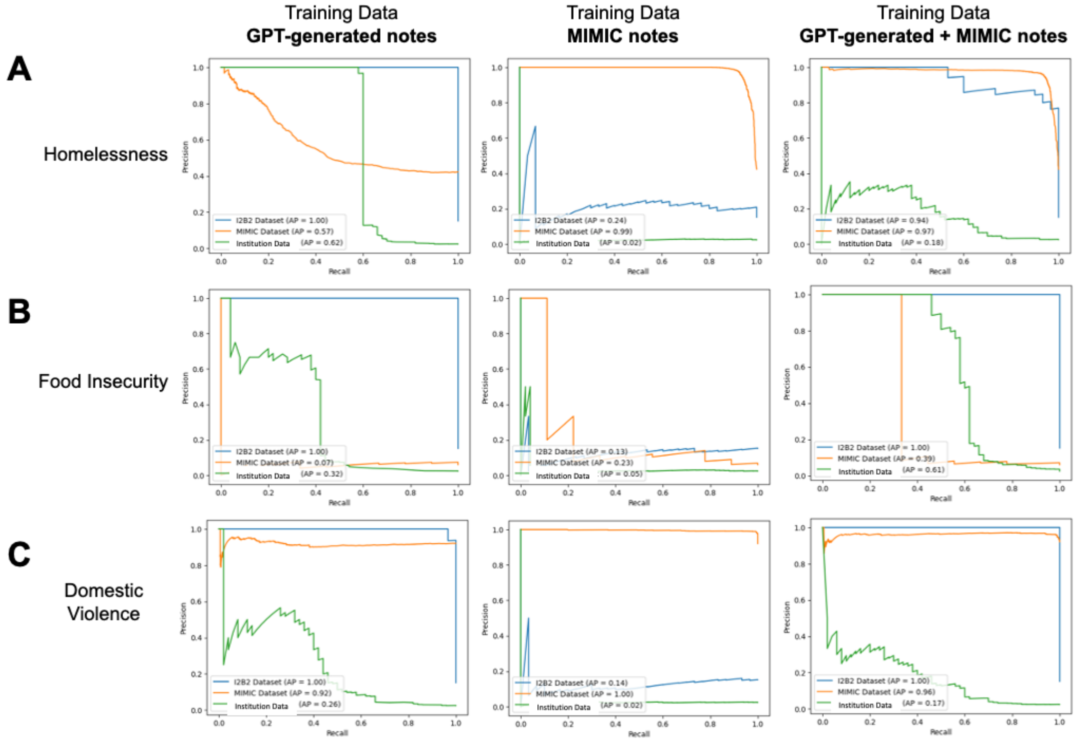

Fig. S2. Precision-recall curves for the large language model-based classifiers using BERT for detecting homelessness, food insecurity, or domestic violence from clinical notes. Each column corresponds to the training set used for the classifier: (1) synthetic notes only, (2) authentic clinical notes from MIMIC-III, and (3) combination of synthetic notes and MIMIC-III. Within each plot, the precision-recall curve is illustrated for when the classifier was validated on three different test sets from i2b2 (synthetic notes), MIMIC-III, and institutional electronic health record notes. Performance is illustrated based on A) homelessness, B) food insecurity, and C) domestic violence. Abbreviations: MIMIC, Medical Information Mart for Intensive Care.

Homelessness

	F1-score	Precision	Recall	Accuracy	Sensitivity	Specificity
**i2B2**						
BERT						
gpt-notes	0.98	0.97	1.00	0.99	1.00	0.99
mimic-notes	0.34	0.21	0.87	0.48	0.87	0.41
gpt+mimic notes	0.89	0.85	0.93	0.96	0.93	0.97
RoBERTa						
gpt-notes	1	1	1.00	1	1.00	1
mimic-notes	0.27	0.16	0.90	0.25	0.90	0.14
gpt+mimic notes	1	1	1.00	1	1.00	1
**MIMIC-3**						
BERT						
gpt-notes	0.02	1.00	0.01	0.58	0.01	1
mimic-notes	0.94	0.94	0.94	0.95	0.94	0.96
gpt+mimic notes	0.94	0.93	0.95	0.95	0.95	0.95
RoBERTa						
gpt-notes	0.003	1	0.00	0.58	0.00	1
mimic-notes	0.92	0.98	0.87	0.93	0.87	0.98
gpt+mimic notes	0.96	0.95	0.98	0.97	0.98	0.96
**Institution Data**						
BERT						
gpt-notes	0.72	1	0.56	0.99	0.56	1
mimic-notes	0.05	0.03	0.80	0.29	0.80	0.28
gpt+mimic notes	0.33	0.32	0.34	0.97	0.34	0.98
RoBERTa						
gpt-notes	0.68	0.9	0.54	0.99	0.54	0.99
mimic-notes	0.07	0.03	0.80	0.43	0.80	0.43
gpt+mimic notes	0.37	0.26	0.60	0.95	0.60	0.96

Food Insecurity

	F1-score	Precision	Recall	Accuracy	Sensitivity	Specificity
**i2B2**						

BERT						
gpt-notes	1	1	1.00	1	1.00	1
mimic-notes	0.23	0.14	0.57	0.42	0.57	0.39
gpt+mimic notes	1	1	1.00	1	1.00	1
RoBERTa						
gpt-notes	0.68	0.94	0.53	0.92	0.53	0.99
mimic-notes	0.26	0.15	1.00	0.15	1.00	0
gpt+mimic notes	1	1	1.00	1	1.00	1
**MIMIC-3**						
BERT						
gpt-notes	0	0.00	0.00	0.93	0.00	0.99
mimic-notes	0.24	0.25	0.22	0.91	0.22	0.96
gpt+mimic notes	0.38	0.43	0.33	0.93	0.33	0.97
RoBERTa						
gpt-notes	0	0	0.00	0.93	0.00	0.99
mimic-notes	0.11	0.06	1.00	0.06	1.00	0
gpt+mimic notes	0.43	0.6	0.33	0.95	0.33	0.99
**Institution Data**						
BERT						
gpt-notes	0.48	0.66	0.38	0.98	0.38	0.99
mimic-notes	0.09	0.04	0.42	0.78	0.42	0.79
gpt+mimic notes	0.64	0.76	0.56	0.98	0.56	0.99
RoBERTa						
gpt-notes	0.1	0.07	0.16	0.93	0.16	0.95
mimic-notes	0.07	0.03	0.80	0.44	0.80	0.43
gpt+mimic notes	0.59	0.92	0.44	0.99	0.44	0.99

Domestic Violence

	F1-score	Precision	Recall	Accuracy	Sensitivity	Specificity
**i2B2**						
BERT						
gpt-notes	0.97	0.94	1.00	0.99	1.00	0.99
mimic-notes	0.24	0.14	0.80	0.23	0.80	0.13
gpt+mimic notes	0.97	0.94	1.00	0.99	1.00	0.99

RoBERTa						
gpt-notes	1	1	1.00	1	1.00	1
mimic-notes	0.26	0.16	0.90	0.25	0.90	0.14
gpt+mimic notes	1	1	1.00	1	1.00	1
**MIMIC-3**						
BERT						
gpt-notes	0.38	0.93	0.24	0.29	0.24	0.79
mimic-notes	0.98	0.99	0.97	0.96	0.97	0.89
gpt+mimic notes	0.97	0.96	0.98	0.94	0.98	0.51
RoBERTa						
gpt-notes	0.003	1	0.00	0.58	0.00	1
mimic-notes	0.92	0.97	0.87	0.93	0.87	0.98
gpt+mimic notes	0.96	0.95	0.98	0.97	0.98	0.96
**Institution Data**						
BERT						
gpt-notes	0.39	0.5	0.32	0.98	0.32	0.99
mimic-notes	0.06	0.03	0.86	0.28	0.86	0.27
gpt+mimic notes	0.29	0.29	0.30	0.96	0.30	0.98
RoBERTa						
gpt-notes	0.68	0.9	0.54	0.99	0.54	0.99
mimic-notes	0.05	0.03	0.86	0.19	0.86	0.18
gpt+mimic notes	0.36	0.26	0.60	0.95	0.60	0.96

Table S1. Performance metrics of language model classifiers (BERT, RoBERTa) for identifying homelessness, food insecurity, and domestic violence from test data.

command_homelessness = """I'm creating a dataset to train an NLP model which can identify 'Social determinants of Health'

when given a medical report of a patient. The determinant which I want to focus on is 'Homelessness'. Give me 100

examples which each at least have 1 to 2 sentences related to the determinant 'Homelessness'. Limit the use of the

phrase 'Homelessness' when possible. Just give the text, don't include the patient name and other information.

Each example should start with keyword 'Determinant Example'.

"""

command_food_insecurity = """I'm creating a dataset to train an NLP model which can identify 'Social determinants of Health'

when given a medical report of a patient. The determinant which I want to focus on is 'Food Insecurity'. Give me 100

examples which each at least have 1 to 2 sentences related to the determinant 'Food Insecurity'. Limit the use of the

phrase 'Food Insecurity' when possible. Just give the text, don't include the patient name and other information.

Each example should start with keyword 'Determinant Example'.

"""

command_domestic_violence = """I'm creating a dataset to train an NLP model which can identify 'Social determinants of Health'

when given a medical report of a patient. The determinant which I want to focus on is 'Domestic Violence'. Give me 100

examples which each at least have 1 to 2 sentences related to the determinant 'Domestic Violence'. Limit the use of the

phrase 'Domestic Violence' when possible. Just give the text, don't include the patient name and other information.

Each example should start with keyword 'Determinant Example'.

"""

command_not_food_insecurty = """I'm creating a dataset to train an NLP model which can identify 'Social determinants of

Health' when given a medical report of a patient. The determinant which I want to focus on is 'Food Insecurity'. Give me

100 examples which each at least have 1 to 2 sentences related to the patient not having issues with 'Food Insecurity'.

Limit the use of the phrase 'Food Insecurity' when possible. Just give the text, don't include the patient name and

other information. Each example should start with keyword 'Determinant Example'.

"""

command_not_homelessness = """I'm creating a dataset to train an NLP model which can identify 'Social determinants of

Health' when given a medical report of a patient. The determinant which I want to focus on is 'Homelessness'. Give me

100 examples which each at least have 1 to 2 sentences related to the patient not having issues with 'Homelessness'.

Limit the use of the phrase 'Homelessness' when possible. Just give the text, don't include the patient name and

other information. Each example should start with keyword 'Determinant Example'.

"""

command_not_domestic_violence = """I'm creating a dataset to train an NLP model which can identify 'Social determinants of

Health' when given a medical report of a patient. The determinant which I want to focus on is 'Domestic Violence'. Give me

100 examples which each at least have 1 to 2 sentences related to the patient not having issues with 'Domestic Violence'.

Limit the use of the phrase 'Domestic Violence' when possible. Just give the text, don't include the patient name and

other information. Each example should start with keyword 'Determinant Example'.

"""

Table S2. ChatGPT prompts used to create synthetic sentences for various social determinants of health.

Determinant Example 1: The patient presented with multiple health issues, including respiratory infections and malnutrition, which are commonly seen among individuals without stable housing.

Determinant Example 2: Lack of affordable housing was a major contributing factor to the patient's current state of homelessness.

Determinant Example 3: The patient reported continuous difficulty accessing regular healthcare due to their transient living situation.

Determinant Example 4: Living on the streets exposed the patient to the harsh weather conditions, resulting in various health problems including hypothermia and frostbite.

Determinant Example 5: Housing instability played a significant role in the patient's overall health decline, as they struggled to adhere to necessary medication and treatment plans.

Determinant Example 6: Homelessness affected the patient's mental health, leading to increased rates of depression, anxiety, and substance abuse.

Determinant Example 7: The lack of a stable living arrangement made it challenging for the patient to maintain regular hygiene, leading to increased risk of infections and skin diseases.

Determinant Example 8: The patient's homeless status prevented them from having a consistent source of nutrition, resulting in chronic malnutrition and weight loss.

Determinant Example 9: The patient's homelessness caused a delay in seeking medical care, resulting in worsened health conditions and more complex treatment needs.

Determinant Example 10: Homelessness increased the patient's vulnerability to violence, resulting in physical injuries and trauma.

Determinant Example 11: Due to their housing instability, the patient had limited access to clean water and sanitation, putting them at higher risk for waterborne diseases.

Determinant Example 12: The patient's health deteriorated due to prolonged exposure to environmental hazards associated with living in abandoned buildings.

Determinant Example 13: The lack of a permanent address hindered the patient's ability to receive timely updates and appointments for medical follow-ups.

Determinant Example 14: The patient experienced increased stress and anxiety due to the constant fear of eviction, exacerbating their existing health conditions.

Determinant Example 15: Homelessness negatively impacted the patient's sleep patterns, leading to chronic fatigue and compromised immune system functioning.

Determinant Example 16: The patient faced challenges in managing chronic illnesses, such as diabetes, due to limited access to refrigeration for medication storage.

Determinant Example 17: Frequent relocation and unstable living situations disrupted the patient's continuity of care, leading to fragmented healthcare services.

Determinant Example 18: Lack of secure housing increased the patient's risk of exposure to infectious diseases prevalent in crowded shelters or living on the streets.

Determinant Example 19: The patient's homelessness hindered their ability to maintain a healthy diet, resulting in nutritional deficiencies and compromised immune system.

Determinant Example 20: The patient's child experienced frequent school changes due to homelessness, impacting their educational attainment and overall well-being.

Determinant Example 21: Homelessness often resulted in social isolation for the patient, leading to increased feelings of loneliness and mental health issues.

Determinant Example 22: The patient's homeless status made it challenging to manage chronic pain conditions, as they had limited access to appropriate pain management resources.

Determinant Example 23: The patient's homelessness increased their vulnerability to substance abuse, as they sought temporary relief from the challenges of their situation.

Determinant Example 24: Homelessness played a significant role in the patient's deteriorating oral health, as access to dental care was limited or non-existent.

Determinant Example 25: Lack of a permanent address prevented the patient from accessing critical social support services, worsening their overall health outcomes.

Determinant Example 26: Homelessness posed additional barriers to the patient's rehabilitation process following surgery, leading to prolonged recovery times.

Determinant Example 27: The patient's frequent exposure to violence and unsanitary living conditions due to homelessness contributed to chronic skin infections.

Determinant Example 28: The patient's lack of stable housing hindered their ability to maintain consistent medication adherence, resulting in poor disease management.

Determinant Example 29: Homelessness increased the patient's susceptibility to mental health crises and psychiatric hospitalizations.

Determinant Example 30: The patient's homeless status limited their access to job opportunities, perpetuating a cycle of insecurity and ongoing health challenges.

Determinant Example 31: The patient faced difficulties in managing their chronic conditions due to limited access to refrigeration for certain medications, compromising treatment efficacy.

Determinant Example 32: Homelessness contributed to the patient's long periods of hunger and nutritional deficiencies, leading to weakened immune system function.

Determinant Example 33: The patient's homelessness was associated with high levels of stress and constant fear for personal safety, negatively impacting their mental well-being.

Determinant Example 34: Frequent exposure to unsanitary and crowded environments due to homelessness increased the patient's risk of contracting infectious diseases.

Determinant Example 35: The patient's lack of secure housing disrupted their ability to establish regular healthcare providers, resulting in fragmented and inadequate care.

Determinant Example 36: Homelessness led to limited access to necessary medical equipment and supplies, hindering the patient's ability to manage chronic conditions appropriately.

Determinant Example 37: The patient's homelessness complicated the management of their chronic pain, as they had limited access to specialized pain management services.

Determinant Example 38: The patient's transient living situation prevented them from receiving timely medical attention, resulting in the progression of preventable health conditions.

Determinant Example 39: Homelessness increased the patient's vulnerability to substance abuse, often serving as a coping mechanism to escape the challenges of their situation.

Determinant Example 40: Lack of stable housing restricted the patient's ability to engage in physical activity and maintain a healthy lifestyle, contributing to various chronic health conditions.

Determinant Example 41: The patient's homeless status led to frequent exposure to extreme weather conditions, resulting in heat stroke and hypothermia.

Determinant Example 42: Homelessness disrupted the patient's sleep patterns and increased their risk of developing sleep disorders, negatively impacting overall well-being.

Determinant Example 43: The patient's homelessness caused a delay in seeking medical care, leading to the advancement of their health conditions and a higher risk of complications.

Determinant Example 44: Housing instability significantly affected the patient's access to prenatal care, increasing their risk of adverse pregnancy outcomes.

Determinant Example 45: The patient's lack of stable housing resulted in limited access to safe and nutritious food, leading to malnutrition and compromised immune system.

Determinant Example 46: Homelessness heightened the patient's exposure to violence and trauma, further exacerbating their mental health challenges.

Determinant Example 47: The patient struggled to manage their asthma effectively due to homelessness, with increased exposure to triggers such as environmental pollutants.

Determinant Example 48: Housing instability played a role in the patient's non-adherence to prescribed medications and treatment plans, leading to worsening health conditions.

Determinant Example 49: The patient's homeless status made it difficult for them to maintain regular healthcare appointments, resulting in delayed diagnosis and intervention.

Determinant Example 50: Homelessness increased the patient's susceptibility to infections and communicable diseases due to the lack of access to proper hygiene facilities.

Determinant Example 51: The patient's homelessness interfered with their ability to engage in self-care practices, contributing to deteriorating health and hygiene.

Determinant Example 52: The patient faced challenges in maintaining a healthy diet due to lack of cooking facilities and limited access to affordable groceries, resulting in malnutrition.

Determinant Example 53: Homelessness exposed the patient to unsanitary living conditions, increasing their risk of contracting infectious diseases such as tuberculosis.

Determinant Example 54: The patient's homelessness hindered their ability to adhere to scheduled medical treatments, resulting in poor management of their chronic conditions.

Determinant Example 55: Lack of stable housing contributed to high levels of stress and anxiety for the patient, negatively impacting their mental well-being.

Determinant Example 56: The patient's homeless status made it difficult to secure stable employment, perpetuating financial insecurity and ongoing challenges to health.

Determinant Example 57: Homelessness restricted the patient's access to appropriate pain management resources, leading to increased suffering and compromised quality of life.

Determinant Example 58: The patient's homelessness increased their vulnerability to substance abuse and dependence as a coping mechanism to escape the challenges of their situation.

Determinant Example 59: Lack of a permanent address hindered the patient's ability to access social support services, preventing them from accessing essential resources for health and well-being.

Determinant Example 60: The patient's homelessness made it difficult for them to adhere to prescribed treatments for chronic diseases, resulting in frequent exacerbations and complications.

Determinant Example 61: Homelessness increased the patient's susceptibility to mental health crises and emergency psychiatric admissions due to the lack of stable housing and support.

Determinant Example 62: The patient's transient living situation exposed them to unsanitary and overcrowded environments, increasing the risk of contracting infectious diseases like hepatitis.

Determinant Example 63: Lack of stable housing hindered the patient's ability to establish a regular source of primary care, leading to fragmented healthcare and limited preventive services.

Determinant Example 64: Homelessness often caused delayed diagnosis and intervention for the patient, resulting in progression of preventable health conditions and poorer outcomes.

Determinant Example 65: The patient's homeless status compromised their ability to access prenatal care, leading to an increased risk of maternal and fetal health complications.

Determinant Example 66: Housing instability resulted in limited access to nutritious food and a higher reliance on unhealthy, processed foods, contributing to the patient's malnutrition.

Determinant Example 67: Homelessness intensified the patient's exposure to violence and trauma, further worsening their mental health conditions, such as post-traumatic stress disorder.

Determinant Example 68: The patient's transient living situation made it challenging to manage their diabetes effectively due to limited access to appropriate dietary options and diabetes self-care resources.

Determinant Example 69: Lack of secure housing led to a constant fear of eviction for the patient, adding to their stress levels and negatively impacting their overall well-being.

Determinant Example 70: The patient's homelessness restricted their ability to engage in regular physical activity, contributing to sedentary behavior and an increased risk of chronic diseases.

Determinant Example 71: Homelessness resulted in limited access to basic hygiene facilities, increasing the patient's risk of skin infections, lice infestation, and other preventable conditions.

Determinant Example 72: The patient's lack of stable housing disrupted their sleep patterns, leading to sleep deprivation, fatigue, and negative impacts on overall health.

Determinant Example 73: The patient's homelessness was associated with delayed medical interventions, resulting in the development of avoidable complications and prolonged recovery times.

Determinant Example 74: Housing instability compromised the patient's ability to access reproductive health services and contraceptive methods, leading to unintended pregnancies and associated health risks.

Determinant Example 75: Homelessness contributed to limited access to safe and nutritious food for the patient, leading to malnutrition and compromised immune system functioning.

Determinant Example 76: The patient's homeless status exposed them to violence and abuse, further exacerbating their mental health struggles, including depression and anxiety.

Determinant Example 77: Lack of secure housing made it challenging for the patient to manage their chronic respiratory conditions, leading to frequent exacerbations and hospitalizations.

Determinant Example 78: Homelessness hindered the patient's ability to adhere to prescribed medications and treatment plans, resulting in poor disease control and avoidable health complications.

Determinant Example 79: The patient's transient living situation limited their access to stable employment and adequate income, perpetuating the cycle of housing instability and ongoing health challenges.

Determinant Example 80: Homelessness increased the patient's susceptibility to substance abuse and addiction as they faced social isolation, unemployment, and coping with the hardships of their situation.

Determinant Example 81: The patient's lack of a permanent address made it difficult to access important social support services, hindering their ability to access vital resources for health and well-being.

Determinant Example 82: Housing instability hindered the patient's ability to adhere to prescribed treatments for chronic diseases, resulting in uncontrolled symptoms and worsening health outcomes.

Determinant Example 83: Homelessness heightened the patient's vulnerability to mental health crises, often requiring emergency psychiatric care due to the lack of stable housing and support.

Determinant Example 84: The patient's transient living situation exposed them to unsanitary and crowded environments, increasing their risk of contracting infectious diseases, such as tuberculosis or respiratory infections.

Determinant Example 85: Lack of stable housing prevented the patient from establishing a consistent source of primary care, leading to fragmented healthcare services and limited access to preventive care measures.

Determinant Example 86: Homelessness often caused delayed diagnosis and intervention for the patient, resulting in the progression of preventable health conditions and poorer health outcomes.

Determinant Example 87: The patient's homeless status compromised their ability to access prenatal care, leading to an increased risk of maternal and fetal health complications.

Determinant Example 88: Housing instability resulted in limited access to nutritious food and an increased reliance on unhealthy, processed foods, contributing to the patient's malnutrition and compromised immune system.

Determinant Example 89: Homelessness intensified the patient's exposure to violence and trauma, further exacerbating their mental health conditions, such as post-traumatic stress disorder.

Determinant Example 90: The patient's transient living situation made it challenging to manage their diabetes effectively due to limited access to appropriate dietary options and diabetes self-care resources.

Determinant Example 91: Lack of secure housing led to a constant fear of eviction for the patient, adding to their stress levels and negatively impacting their overall well-being.

Determinant Example 92: The patient's homelessness restricted their ability to engage in regular physical activity, contributing to sedentary behavior and an increased risk of chronic diseases.

Determinant Example 93: Homelessness resulted in limited access to basic hygiene facilities, increasing the patient's risk of skin infections, lice infestation, and other preventable conditions.

Determinant Example 94: The patient's lack of stable housing disrupted their sleep patterns, leading to sleep deprivation, fatigue, and negative impacts on overall health.

Determinant Example 95: The patient's homelessness was associated with delayed medical interventions, resulting in the development of avoidable complications and prolonged recovery times.

Determinant Example 96: Housing instability compromised the patient's ability to access reproductive health services and contraceptive methods, leading to unintended pregnancies and associated health risks.

Determinant Example 97: Homelessness contributed to limited access to safe and nutritious food for the patient, leading to malnutrition and compromised immune system functioning.

Determinant Example 98: The patient's homeless status exposed them to violence and abuse, further exacerbating their mental health struggles, including depression and anxiety.

Determinant Example 99: Lack of secure housing made it challenging for the patient to manage their chronic respiratory conditions, leading to frequent exacerbations and hospitalizations.

Determinant Example 100: Homelessness hindered the patient's ability to adhere to prescribed medications and treatment plans, resulting in poor disease control and avoidable health complications.

Table S3. ChatGPT-generated sentences for stating presence of homelessness

Determinant Example 1: The patient has a stable housing situation with a permanent address.

Determinant Example 2: The patient lives in a safe and secure home.

Determinant Example 3: Housing instability is not a concern for the patient as they have a reliable place to live.

Determinant Example 4: The individual has a fixed address, eliminating any housing uncertainty.

Determinant Example 5: The patient reports having a supportive and stable living environment.

Determinant Example 6: The absence of housing challenges allows the patient to focus on their health needs.

Determinant Example 7: The patient has a physical residence that provides them with a sense of belonging and security.

Determinant Example 8: Housing insecurity is not a factor in this case, enabling the patient to prioritize their overall well-being.

Determinant Example 9: The individual is not facing housing instability and can allocate their resources towards maintaining good health.

Determinant Example 10: The patient has a permanent residence, fostering a sense of stability and emotional well-being.

Determinant Example 11: The absence of homelessness issues allows the patient to access consistent healthcare services.

Determinant Example 12: Having a home eliminates any potential risk factors associated with homelessness.

Determinant Example 13: The patient has a reliable and consistent housing situation which positively impacts their health outcomes.

Determinant Example 14: The individual resides in a home that aligns with their social and emotional needs.

Determinant Example 15: Housing stability enables the patient to engage in activities that promote their physical and mental well-being.

Determinant Example 16: The patient experiences security and comfort within their current housing arrangement.

Determinant Example 17: The lack of housing instability allows the patient to focus on maintaining a healthy lifestyle.

Determinant Example 18: The individual is not burdened by the stressors typically associated with homelessness.

Determinant Example 19: The patient has the opportunity to create a nurturing environment at their permanent residence.

Determinant Example 20: Access to stable housing positively impacts the patient's overall quality of life.

Determinant Example 21: The patient does not face challenges related to housing insecurity, which positively influences their health.

Determinant Example 22: The absence of housing issues provides a foundation for the patient's overall well-being.

Determinant Example 23: The individual's housing situation does not pose any immediate health risks.

Determinant Example 24: The patient enjoys a secure and stable living arrangement, devoid of any housing concerns.

Determinant Example 25: The absence of homelessness allows the patient to focus on their medical treatment without additional stressors.

Determinant Example 26: The individual has a permanent address, facilitating consistent healthcare access.

Determinant Example 27: The patient resides in a supportive living environment, contributing to their overall health stability.

Determinant Example 28: The absence of housing insecurity reduces potential barriers to healthcare services.

Determinant Example 29: The patient's housing situation does not impact their ability to seek appropriate medical care.

Determinant Example 30: Stable housing provides the patient with a foundation for managing their health effectively.

Determinant Example 31: Housing stability allows the patient to maintain their everyday routines and prioritize their health.

Determinant Example 32: The individual's housing arrangement offers them a safe and comfortable place to recover from illnesses.

Determinant Example 33: The patient is not faced with the additional challenges associated with housing instability.

Determinant Example 34: The absence of homelessness supports the patient's ability to adapt to their healthcare regimen.

Determinant Example 35: The individual's housing security promotes their emotional well-being, positively impacting their overall health.

Determinant Example 36: The patient can rely on a stable home environment as they navigate their health journey.

Determinant Example 37: Housing stability fosters a sense of belonging and assists in building a strong support system for the patient.

Determinant Example 38: The individual's housing situation does not hinder their access to essential health resources.

Determinant Example 39: The patient does not experience chronic stressors associated with housing insecurity, which facilitates their health management.

Determinant Example 40: Stable housing eliminates a significant determinant impacting the patient's health outcomes.

Determinant Example 41: The patient has a reliable living situation, ensuring a consistent environment for improving their overall health.

Determinant Example 42: The absence of homelessness allows the patient to focus on their occupational and educational goals.

Determinant Example 43: The individual's housing stability promotes a positive attitude towards healthcare engagement.

Determinant Example 44: The patient's consistent housing arrangement is conducive to their well-being and health maintenance.

Determinant Example 45: The patient does not face challenges related to finding temporary or emergency shelter, promoting a sense of security.

Determinant Example 46: The absence of housing instability contributes to the patient's overall social connectedness and support network.

Determinant Example 47: The individual resides in a nurturing home environment, promoting a positive trajectory in their healthcare journey.

Determinant Example 48: Housing stability fosters a sense of safety and trust, positively impacting the patient's mental health.

Determinant Example 49: The patient has access to necessary utilities and amenities, supporting their health-related needs.

Determinant Example 50: The absence of homelessness allows the patient to actively participate in their healthcare decision-making process.

Determinant Example 51: The patient resides in a community where housing assistance programs are readily available, ensuring a stable living arrangement.

Determinant Example 52: Housing stability contributes to the patient's ability to establish and maintain healthy lifestyle behaviors.

Determinant Example 53: The individual's secure housing situation eliminates potential barriers to engaging in preventive healthcare.

Determinant Example 54: The patient has a permanent home that allows for the development of social connections and engagement in community resources.

Determinant Example 55: The absence of homelessness promotes a more optimistic outlook on the patient's health prospects.

Determinant Example 56: The patient's housing stability provides a foundation for managing and overcoming health challenges.

Determinant Example 57: The individual's consistent housing situation fosters a sense of pride and personal fulfillment.

Determinant Example 58: The patient's access to a physical home positively contributes to their overall health and well-being.

Determinant Example 59: The absence of housing unpredictability facilitates the patient's adherence to treatment plans.

Determinant Example 60: The patient's stable living arrangement reduces the likelihood of experiencing health disruptions due to housing issues.

Determinant Example 61: Housing stability minimizes the patient's exposure to environmental health risks commonly associated with homelessness.

Determinant Example 62: The individual's housing security supports their ability to manage chronic conditions effectively.

Determinant Example 63: The patient has a consistent and secure residence that enhances their self-care practices.

Determinant Example 64: The absence of homelessness allows the patient to focus on building resilience and capacity for self-management.

Determinant Example 65: The patient does not face the challenges of finding temporary or emergency accommodations, allowing for uninterrupted healthcare.

Determinant Example 66: The individual's housing stability contributes to their sense of personal agency and control in managing their health.

Determinant Example 67: The patient's housing situation promotes a healthy living environment conducive to recovery and rehabilitation.

Determinant Example 68: The absence of housing instability enables the patient to establish stable relationships with healthcare providers.

Determinant Example 69: The patient's consistent housing arrangement fosters a sense of empowerment and self-advocacy in their healthcare journey.

Determinant Example 70: Housing stability minimizes disruptions in the patient's healthcare access and continuity of care.

Determinant Example 71: The individual's safe and secure housing situation supports their overall health and well-being.

Determinant Example 72: The patient's housing stability creates opportunities for social integration and participation in community activities.

Determinant Example 73: The absence of homelessness ensures the patient's ability to focus on long-term health goals rather than immediate survival needs.

Determinant Example 74: The patient's housing security contributes to their mental and emotional resilience during times of health challenges.

Determinant Example 75: The individual's consistent housing arrangement fosters a sense of belonging and social support, positively influencing their health.

Determinant Example 76: Housing stability eliminates the risk of exposure to unsafe living conditions that can compromise the patient's health.

Determinant Example 77: The patient resides in a neighborhood known for its adequate housing options and low rates of homelessness.

Determinant Example 78: The absence of housing issues allows the patient to allocate resources towards maintaining good health practices.

Determinant Example 79: The individual does not experience the stressors associated with housing insecurity, removing potential barriers to their health.

Determinant Example 80: Housing stability positively impacts the patient's overall health literacy and ability to navigate healthcare systems.

Determinant Example 81: The patient's physical residence supports their involvement in community activities that promote holistic well-being.

Determinant Example 82: The absence of homelessness allows the patient to focus on personal growth and achieving self-defined health goals.

Determinant Example 83: The individual's stable housing environment facilitates consistent medication adherence and preventive care.

Determinant Example 84: The patient's housing stability allows them to access healthcare services without additional logistical challenges.

Determinant Example 85: Housing security contributes to the patient's sense of safety and emotional well-being, positively affecting their health status.

Determinant Example 86: The absence of housing instability provides the patient with a reliable and comfortable place to recover from health setbacks.

Determinant Example 87: The individual's housing situation does not hinder their ability to engage in physical activities that promote health.

Determinant Example 88: The patient's stable home environment fosters a sense of normalcy, positively influencing their mental health outcomes.

Determinant Example 89: Housing stability facilitates the patient's ability to engage in health promotion activities and preventive screening.

Determinant Example 90: The absence of homelessness allows the patient to actively participate in skill-building and educational opportunities related to health management.

Determinant Example 91: The patient does not have to rely on temporary shelters or emergency accommodations, ensuring a consistent healthcare journey.

Determinant Example 92: The individual's secure housing arrangement supports the development of a routine that promotes self-care and health maintenance.

Determinant Example 93: The patient's housing stability contributes to their ability to access social resources that positively impact their well-being.

Determinant Example 94: Housing security enables the patient to establish trust and long-term relationships with healthcare providers.

Determinant Example 95: The absence of housing insecurity allows the patient to invest in long-term health-related goals and aspirations.

Determinant Example 96: The patient's stable home environment fosters a sense of peace and stability, reducing stressors that can impact health negatively.

Determinant Example 97: Housing stability facilitates the patient's ability to engage in self-management practices and maintain a set healthcare routine.

Determinant Example 98: The individual's consistent housing situation encourages a sense of hope and optimism about their health prospects.

Determinant Example 99: The patient's access to stable housing supports their community integration and engagement, vital for holistic health.

Determinant Example 100: The absence of homelessness minimizes disruptions in the patient's healthcare journey, promoting continuity of care.

Table S4. ChatGPT-generated sentences for negating presence of homelessness

Determinant Example 1: Patient presents with a history of low income and inadequate access to affordable, nutritious food options.

Determinant Example 2: The patient's report reveals concerns about food scarcity and lack of regular meals due to financial constraints.

Determinant Example 3: The medical records indicate that the patient is struggling with food access, leading to skipped meals and compromised nutrition.

Determinant Example 4: The patient's health issues can be partly attributed to their inability to afford quality groceries and maintain a healthy diet.

Determinant Example 5: The report highlights the patient's reliance on food assistance programs as a result of limited financial resources for consistent meal provision.

Determinant Example 6: Inadequate access to fresh fruits and vegetables contributes to the patient's poor health outcomes, indicating food insecurity.

Determinant Example 7: Financial constraints reported by the patient lead to compromised food choices, impacting their overall nutrition and health status.

Determinant Example 8: The patient's medical report highlights their struggle with hunger due to insufficient income and lack of food resources.

Determinant Example 9: The patient's nutritional deficiencies suggest a pattern of food insecurity resulting from limited financial means.

Determinant Example 10: The report indicates that the patient frequently experiences hunger and has difficulties obtaining nutritious food due to financial limitations.

Determinant Example 11: The patient's health conditions can be partly attributed to their long-term struggle with access to an adequate and nutritious food supply.

Determinant Example 12: The medical records reveal that the patient often reallocates funds meant for other necessities to ensure a steady food supply, indicating food insecurity.

Determinant Example 13: The patient's inability to consistently afford nutritious meals contributes to their compromised immune system and overall health.

Determinant Example 14: The report highlights the patient's reliance on food pantries and community organizations for sustenance, indicating food insecurity.

Determinant Example 15: The patient's weight fluctuations and signs of malnutrition align with the profile of an individual experiencing food insecurity.

Determinant Example 16: The medical report suggests that the patient's recurring health issues may be exacerbated by their limited access to quality, affordable food.

Determinant Example 17: The patient's financial constraints result in irregular meal patterns and inadequate nutrition, indicating food insecurity.

Determinant Example 18: The records show that the patient's inability to consistently afford food compromises their ability to manage chronic conditions effectively.

Determinant Example 19: The patient's significant weight loss is indicative of food insecurity, where they struggle to maintain an adequate caloric intake.

Determinant Example 20: The report highlights the patient's reliance on food banks, reflecting their insufficient income to consistently cover their nutritional needs.

Determinant Example 21: The patient's health issues can be partly attributed to their lack of access to affordable, nutritious food due to financial difficulties.

Determinant Example 22: The medical records indicate that the patient's food insecurity has led to skipped meals and an unbalanced diet.

Determinant Example 23: The patient's inability to obtain adequate food contributes to their increased risk of developing chronic diseases.

Determinant Example 24: The report reveals that the patient experiences anxiety related to their uncertain food supply, affecting their well-being.

Determinant Example 25: The patient's limited financial resources result in food choices that are high in calories but low in nutritional value.

Determinant Example 26: The records suggest that the patient's frequent reliance on fast food and unhealthy snacks is due to limited access to affordable, nutritious options.

Determinant Example 27: The patient's ongoing struggle with food insecurity exacerbates their mental health issues, leading to a vicious cycle of poor well-being.

Determinant Example 28: The medical report indicates that the patient's lack of access to nutritious food contributes to delayed healing and prolonged recovery.

Determinant Example 29: The patient's difficulties affording groceries and fresh produce result in an imbalanced diet lacking essential nutrients.

Determinant Example 30: The report highlights the patient's constant worry about their next meal and the impact it has on their overall health.

Determinant Example 31: The patient's lab results indicate deficiencies in vital nutrients, suggesting a link to food insecurity and compromised dietary intake.

Determinant Example 32: The records show that the patient frequently experiences hunger due to an inadequate budget for food, impacting their overall well-being.

Determinant Example 33: The patient's inability to consistently afford nutritious meals hinders their ability to manage chronic conditions and achieve optimal health outcomes.

Determinant Example 34: The medical report suggests that the patient's limited food access leads to reliance on cheaper, highly processed food options that negatively impact their health.

Determinant Example 35: The patient's persistent food insecurity results in irregular meal patterns and inadequate intake of essential vitamins and minerals.

Determinant Example 36: The report highlights the patient's reliance on food stamps as their primary source of sustenance, indicating ongoing food insecurity.

Determinant Example 37: The patient's inability to afford a diverse range of foods impacts their dietary quality and increases the risk of nutrient deficiencies.

Determinant Example 38: The medical records reveal that the patient's insufficient income leads to forgoing food purchases, resulting in meals with limited nutritional value.

Determinant Example 39: The patient's reliance on low-cost, high-calorie foods underscores their ongoing struggle with food insecurity.

Determinant Example 40: The report suggests that the patient's limited access to fresh fruits and vegetables contributes to their suboptimal health outcomes.

Determinant Example 41: The patient's financial constraints prevent them from purchasing sufficient quantities of meat, dairy, and other protein sources necessary for a balanced diet.

Determinant Example 42: The medical report highlights the patient's reliance on food donations to make ends meet, indicating underlying food insecurity.

Determinant Example 43: The patient's weight gain can be attributed to their tendency to consume cheaper, energy-dense foods due to food insecurity.

Determinant Example 44: The records indicate that the patient's constant worry about accessing food negatively impacts their mental well-being and stress levels.

Determinant Example 45: The patient's lack of consistent access to nutritious food compromises their immune system, making them more susceptible to infections.

Determinant Example 46: The report suggests that the patient's food insecurity perpetuates a cycle of poor health outcomes, limited resources, and ongoing medical issues.

Determinant Example 47: The patient's low income forces them to prioritize other essential expenses over food, leading to compromised nutritional intake.

Determinant Example 48: The medical records reveal that the patient's food insecurity hinders their ability to adhere to prescribed dietary restrictions.

Determinant Example 49: The patient's inability to purchase fresh produce regularly indicates a limited budget for nutritious food, resulting in compromised health.

Determinant Example 50: The report highlights the patient's reliance on processed and packaged foods due to limited time and financial resources, contributing to an unhealthy diet.

Determinant Example 51: The patient's struggle with food insecurity is evident in their frequent visits to food assistance programs and reliance on community resources.

Determinant Example 52: The records suggest that the patient's lack of access to affordable, nutritious food contributes to their weight-related health issues.

Determinant Example 53: The patient's limited financial means result in poor food choices, often opting for cheap, unhealthy options that lack essential nutrients.

Determinant Example 54: The medical report indicates that the patient experiences feelings of shame and embarrassment associated with their reliance on food aid due to food insecurity.

Determinant Example 55: The patient's compromised nutritional status is linked to their recurring experience of food scarcity and uncertainty about their next meal.

Determinant Example 56: The report reveals that the patient's household income is insufficient to consistently provide nutritious meals, resulting in food insecurity.

Determinant Example 57: The patient's inability to afford regular meals and uncertainty about food availability contributes to their chronic stress levels.

Determinant Example 58: The medical records highlight the patient's reliance on unhealthy, high-calorie foods due to limited access to affordable, nutritious alternatives.

Determinant Example 59: The patient's weight management difficulties can be partly attributed to their irregular food intake caused by food insecurity.

Determinant Example 60: The report suggests that the patient's food insecurity is further exacerbated by living in a food desert with limited grocery options.

Determinant Example 61: The patient's suboptimal dietary choices are driven by their low income, leading to a heightened risk of chronic diseases associated with food insecurity.

Determinant Example 62: The medical report indicates that the patient experiences anxiety and depression related to their uncertain food supply, negatively impacting their mental health.

Determinant Example 63: The patient's inability to access healthy food options prevents them from adequately addressing their nutritional needs and maintaining overall well-being.

Determinant Example 64: The records suggest that the patient's prolonged food insecurity affects their dietary diversity, resulting in inadequate intake of essential vitamins and minerals.

Determinant Example 65: The patient's limited income leads to skipped meals and reduced portion sizes, resulting in compromised nutrition and increased vulnerability to illness.

Determinant Example 66: The report highlights the patient's reliance on cheap, highly processed foods due to financial constraints, contributing to their poor health outcomes.

Determinant Example 67: The patient's inadequate access to nutritious food options leads to an imbalanced diet lacking in essential macronutrients and micronutrients.

Determinant Example 68: The medical records reveal that the patient's inability to afford a consistent food supply leads to frequent periods of hunger and malnourishment.

Determinant Example 69: The patient's food insecurity manifests in their persistent concern about running out of food and inability to afford quality groceries.

Determinant Example 70: The report suggests that the patient's lack of access to affordable, fresh food is linked to their increased risk of developing diet-related chronic conditions.

Determinant Example 71: The patient's limited budget for food compromises their ability to prioritize nutrition, leading to an increased likelihood of vitamin and mineral deficiencies.

Determinant Example 72: The records indicate that the patient's ongoing food insecurity exacerbates their existing health conditions and inhibits their ability to achieve optimal wellness.

Determinant Example 73: The patient's reliance on charitable organizations and food banks demonstrates their inability to consistently afford an adequate food supply.

Determinant Example 74: The medical report highlights the patient's constant trade-offs between essential expenses and food purchases, resulting in compromised nutrition.

Determinant Example 75: The patient's financial struggles restrict their ability to purchase fresh produce, contributing to a micronutrient-poor diet lacking in essential vitamins and minerals.

Determinant Example 76: The report suggests that the patient's low income perpetuates a cycle of food insecurity and limited access to health-promoting food choices.

Determinant Example 77: The patient's reduced ability to afford a nutritious diet leads to increased reliance on low-cost, energy-dense foods associated with poor health outcomes.

Determinant Example 78: The records reveal that the patient's food insecurity impacts their children's nutritional status, putting them at risk of developmental issues.

Determinant Example 79: The patient's constant worry about food availability and the need to stretch limited resources affects their mental well-being and overall quality of life.

Determinant Example 80: The medical report indicates that the patient's food insecurity results in compromised immune function, leading to frequent infections and delayed healing.

Determinant Example 81: The patient's limited financial means force them to make trade-offs between medications and food, negatively impacting their overall health management.

Determinant Example 82: The report emphasizes the patient's reliance on food assistance programs as a coping strategy for their ongoing food insecurity.

Determinant Example 83: The patient's inability to afford nutritious meals contributes to their increased likelihood of experiencing diet-related chronic diseases, such as diabetes and hypertension.

Determinant Example 84: The records suggest that the patient's restricted access to healthy food sources contributes to their weight gain and obesity-related health issues.

Determinant Example 85: The patient's food insecurity is evident in their fluctuating weight, indicating periods of inadequate food supply and subsequent compensatory overeating.

Determinant Example 86: The report highlights the patient's anxiety and depression related to their inability to consistently afford nutritious meals, impacting their mental health.

Determinant Example 87: The patient's compromised access to healthy food options results in a diet primarily composed of highly processed foods lacking in essential nutrients.

Determinant Example 88: The medical records reveal that the patient frequently skips meals or eats smaller portions due to financial constraints, resulting in inadequate nutrition.

Determinant Example 89: The patient's limited income restricts their ability to buy a variety of food, leading to a monotonous, less nutrient-rich diet.

Determinant Example 90: The report suggests that the patient's food insecurity is further worsened by transportation barriers, limiting their access to grocery stores and fresh produce.

Determinant Example 91: The patient's unpredictable access to food and limited resources contribute to stress and negatively impact their ability to manage their health conditions effectively.

Determinant Example 92: The records indicate that the patient's food scarcity results in dietary patterns dominated by inexpensive, energy-dense foods with low nutritional value.

Determinant Example 93: The patient's reliance on fast food and convenience store options due to limited financial means contributes to their increased risk of obesity and related health issues.

Determinant Example 94: The medical report highlights the patient's constant trade-offs between food and housing expenses, leading to compromised nutritional intake.

Determinant Example 95: The patient's limited food options and insufficient income hinder their ability to adopt and maintain a balanced diet necessary for optimal health.

Determinant Example 96: The report suggests that the patient's food insecurity contributes to their higher likelihood of experiencing mental health disorders, such as depression and anxiety.

Determinant Example 97: The patient's inability to consistently afford nutritious meals leads to compromised growth and development, particularly concerning in pediatric cases.

Determinant Example 98: The records reveal that the patient's food insecurity is compounded by living in an area with limited access to grocery stores and fresh, healthy food options.

Determinant Example 99: The patient's inadequate food supply affects their medication adherence and ability to manage chronic conditions effectively, perpetuating health issues.

Determinant Example 100: The report highlights the patient's reliance on inexpensive, calorie-dense foods due to financial constraints, leading to chronic malnutrition and associated health complications.

Table S5. ChatGPT-generated sentences for presence of food insecurity

Determinant Example: The patient reports having enough food to eat and does not mention any concerns about access or affordability.
Determinant Example: The patient's medical report does not indicate any signs of hunger or financial struggling to afford food.
Determinant Example: The patient mentions being able to consistently access and afford nutritious food without any issues.
Determinant Example: The patient does not express any difficulties related to obtaining or affording food in their medical history.
Determinant Example: There are no indications of food insecurity or related concerns in the patient's medical record.
Determinant Example: The patient's medical report does not mention any challenges or worries regarding accessing nutritious food.
Determinant Example: There is no evidence of food insecurity in the patient's history or current health status.
Determinant Example: The patient states that they have reliable access to an adequate amount of food and do not express any concerns.
Determinant Example: The medical report does not indicate any signs of food insecurity or inadequate access to nourishing meals.
Determinant Example: The patient reports having a stable source of income that allows them to purchase food without difficulty.
Determinant Example: There are no recorded instances of the patient experiencing hunger or struggling to afford food in their medical record.
Determinant Example: The patient mentions regularly eating three meals a day and does not express any worries about food availability or affordability.
Determinant Example: The medical report shows no indications of the patient facing food insecurity or issues with accessing nutritious meals.
Determinant Example: The patient's history does not reflect any instances of hunger or financial constraints preventing them from obtaining food.
Determinant Example: The patient states that they have access to fresh and healthy food options in their local area.
Determinant Example: There are no documented concerns or incidents indicating food insecurity in the patient's medical history.
Determinant Example: The medical report does not mention any difficulties in the patient's ability to afford or acquire the necessary food for their well-being.
Determinant Example: The patient confirms having consistent access to a variety of nutritious foods without facing any obstacles.
Determinant Example: There are no signs of food scarcity or insufficient access to nourishing meals in the patient's medical records.
Determinant Example: The patient describes their current food situation as stable without any incidences of food insecurity to report.
Determinant Example: The medical report shows no indications of the patient experiencing hunger or struggling with food affordability.
Determinant Example: The patient's history does not mention any challenges related to food access or affordability.
Determinant Example: The patient states that they have never experienced food insecurity or struggled to maintain an adequate diet.
Determinant Example: The medical report does not contain any notes regarding the patient's food consumption, suggesting no presence of food insecurity.
Determinant Example: The patient confirms never going without food or experiencing any issues related to food availability or affordability.
Determinant Example: There are no recorded concerns or incidents suggesting that the patient has faced food insecurity at any point.
Determinant Example: The medical report does not indicate any obstacles or hardships related to the patient's ability to obtain nutritious food.
Determinant Example: The patient expresses having a stable and predictable source of food without experiencing any disruptions.
Determinant Example: The patient's medical history contains no instances of food insecurity or struggles with obtaining adequate nutrition.
Determinant Example: The medical report does not mention any instances of the patient being unable to afford or access food.
Determinant Example: The patient confirms never experiencing hunger or difficulties related to obtaining nourishing meals.

Determinant Example: There are no indications or mentions of food insecurity in the patient's medical record.
Determinant Example: The medical report shows no signs of the patient facing challenges in acquiring nutritious food.
Determinant Example: The patient's history does not reflect any instances of food scarcity or difficulties with affording meals.
Determinant Example: The patient states that they have never faced food insecurity or struggled to maintain a constant food supply.
Determinant Example: The medical report does not contain any remarks regarding the patient's food security or access to nutrition.
Determinant Example: The patient confirms having consistent access to a wide range of nutritious food without any disruptions.
Determinant Example: There are no documented concerns or incidents suggesting the patient has experienced food insecurity.
Determinant Example: The medical report does not mention any difficulties related to the patient's ability to obtain or afford adequate food.
Determinant Example: The patient expresses having a stable income that allows them to purchase food without any financial strain.
Determinant Example: The patient's medical history contains no indications of food insecurity or issues regarding food affordability.
Determinant Example: The medical report does not mention any obstacles or hardships related to the patient's access to nourishing meals.
Determinant Example: The patient reports being able to consistently afford and obtain healthy food options without difficulty.
Determinant Example: There are no recorded concerns or incidents suggesting the patient has faced food insecurity at any point in their life.
Determinant Example: The medical report does not indicate any signs of the patient experiencing hunger or struggling with food accessibility.
Determinant Example: The patient's history does not mention any challenges related to food access or financial constraints preventing them from obtaining meals.
Determinant Example: The patient states that they have never experienced food insecurity or gone without an adequate diet.
Determinant Example: The medical report does not contain any remarks regarding the patient facing difficulties in obtaining or affording food.
Determinant Example: The patient confirms never experiencing hunger or any issues related to obtaining nutritious food.
Determinant Example: There are no indications or mentions of food insecurity in the patient's medical records.
Determinant Example: The medical report shows no indications of the patient facing challenges in acquiring or affording nourishing meals.
Determinant Example: The patient's history does not reflect any instances of food scarcity or struggles with food affordability.
Determinant Example: The patient states that they have never faced food insecurity or struggled to maintain an adequate food supply.
Determinant Example: The medical report does not mention any obstacles or hardships related to the patient's access to nutritious food.
Determinant Example: The patient expresses having a stable and reliable source of food without any disruptions or difficulties.
Determinant Example: There are no recorded concerns or incidents indicating the patient has experienced food insecurity.
Determinant Example: The medical report does not contain any remarks regarding the patient's food security or any difficulties in accessing nutrition.
Determinant Example: The patient confirms having consistent access to a diverse range of nutritious food without any disruptions or hesitations.
Determinant Example: There are no documented concerns or incidents suggesting the patient has faced food insecurity.
Determinant Example: The medical report does not mention any difficulties related to the patient's ability to obtain or afford adequate food.
Determinant Example: The patient expresses having a stable financial situation that allows them to purchase food without any financial strain or constraints.
Determinant Example: The patient's medical history contains no indications of food insecurity or issues regarding food accessibility.
Determinant Example: The medical report does not mention any obstacles or hardships related to the patient's access to nourishing meals or financial difficulties related to food.

Determinant Example: The patient reports being able to consistently afford and obtain a wide range of healthy food options without difficulty or inconvenience.
Determinant Example: There are no recorded concerns or incidents suggesting the patient has faced food insecurity at any point in their life.
Determinant Example: The medical report does not indicate any signs of the patient experiencing hunger or struggling with food accessibility or affordability.
Determinant Example: The patient's history does not mention any challenges related to food access or financial constraints preventing them from obtaining and enjoying meals without any difficulty.
Determinant Example: The patient states that they have never experienced food insecurity or gone without an adequate diet due to financial issues or scarcity of food.
Determinant Example: The medical report does not contain any remarks regarding the patient facing difficulties in obtaining or affording food, suggesting a reliable source of nutrition throughout their lifetime.
Determinant Example: The patient confirms never experiencing hunger or any issues related to obtaining or affording nutritious food, reflecting a consistent food security status.
Determinant Example: There are no indications or mentions of food insecurity in the patient's medical records, indicating a stable and secure food environment for the individual.
Determinant Example: The medical report shows no indications of the patient facing challenges in acquiring or affording nourishing meals, suggesting a consistently adequate diet.
Determinant Example: The patient's history does not reflect any instances of food scarcity or struggles with food affordability, indicating a consistent state of food security.
Determinant Example: The patient states that they have never faced food insecurity or struggled to maintain an adequate food supply, reflecting a secure food situation for the individual.
Determinant Example: The medical report does not mention any obstacles or hardships related to the patient's access to nutritious food, indicating an uninterrupted availability of nourishing meals.
Determinant Example: The patient expresses having a stable and reliable source of food without any disruptions or difficulties, underscoring consistent food security.
Determinant Example: There are no recorded concerns or incidents indicating the patient has experienced food insecurity, suggesting a continuously secure food environment.
Determinant Example: The medical report does not contain any remarks regarding the patient's food security or any difficulties in accessing nutrition, highlighting a reliable food supply.
Determinant Example: The patient confirms having consistent access to a diverse range of nutritious food without any disruptions or hesitations, pointing towards a maintained state of food security.
Determinant Example: There are no documented concerns or incidents suggesting the patient has faced food insecurity, reinforcing a long-standing and ongoing food security.
Determinant Example: The medical report does not mention any difficulties related to the patient's ability to obtain or afford adequate food, indicating a stable financial condition to maintain food security.
Determinant Example: The patient expresses having a stable financial situation that allows them to purchase food without any financial strain or constraints, safeguarding food security for the individual.
Determinant Example: The patient's medical history contains no indications of food insecurity or issues regarding food accessibility, affirming a sustained access to an ample variety of nutritious meals.
Determinant Example: The medical report does not mention any obstacles or hardships related to the patient's access to nourishing meals or financial difficulties related to food, signifying uninterrupted food security over time.
Determinant Example: The patient reports being able to consistently afford and obtain a wide range of healthy food options without difficulty or inconvenience, signifying a continued food security status.
Determinant Example: There are no recorded concerns or incidents suggesting the patient has faced food insecurity at any point in their life, underscoring a prolonged food-secure environment.
Determinant Example: The medical report does not indicate any signs of the patient experiencing hunger or struggling with food accessibility or affordability, attesting to persistent food security.
Determinant Example: The patient's history does not mention any challenges related to food access or financial constraints preventing them from obtaining and enjoying meals without any difficulty, validating sustained food security.
Determinant Example: The patient states that they have never experienced food insecurity or gone without an adequate diet due to financial issues or scarcity of food, reinforcing their ongoing food security status.
Determinant Example: The medical report does not contain any remarks regarding the patient facing difficulties in obtaining or affording food, suggesting a reliable source of nutrition throughout their lifetime.
Determinant Example: The patient confirms never experiencing hunger or any issues related to obtaining or affording nutritious food, reflecting a consistent food security status.
Determinant Example: There are no indications or mentions of food insecurity in the patient's medical records, indicating a stable and secure food environment for the individual.

Determinant Example: The medical report shows no indications of the patient facing challenges in acquiring or affording nourishing meals, suggesting a consistently adequate diet.

Determinant Example: The patient's history does not reflect any instances of food scarcity or struggles with food affordability, indicating a consistent state of food security.

Determinant Example: The patient states that they have never faced food insecurity or struggled to maintain an adequate food supply, reflecting a secure food situation for the individual.

Determinant Example: The medical report does not mention any obstacles or hardships related to the patient's access to nutritious food, indicating an uninterrupted availability of nourishing meals.

Determinant Example: The patient expresses having a stable and reliable source of food without any disruptions or difficulties, underscoring consistent food security.

Determinant Example: There are no recorded concerns or incidents indicating the patient has experienced food insecurity, suggesting a continuously secure food environment.

Determinant Example: The medical report does not contain any remarks regarding the patient's food security or any difficulties in accessing nutrition, highlighting a reliable food supply.

Determinant Example: The patient confirms having consistent access to a diverse range of nutritious food without any disruptions or hesitations, pointing towards a maintained state of food security.

Determinant Example: There are no documented concerns or incidents suggesting the patient has faced food insecurity, reinforcing a long-standing and ongoing food security.

Determinant Example: The medical report does not mention any difficulties related to the patient's ability to obtain or afford adequate food, indicating a stable financial condition to maintain food security.

Determinant Example: The patient expresses having a stable financial situation that allows them to purchase food without any financial strain or constraints, safeguarding food security for the individual.

Determinant Example: The patient's medical history contains no indications of food insecurity or issues regarding food accessibility, affirming a sustained access to an ample variety of nutritious meals.

Determinant Example: The medical report does not mention any obstacles or hardships related to the patient's access to nourishing meals or financial difficulties related to food, signifying uninterrupted food security over time.

Determinant Example: The patient reports being able to consistently afford and obtain a wide range of healthy food options without difficulty or inconvenience, signifying a continued food security status.

Determinant Example: There are no recorded concerns or incidents suggesting the patient has faced food insecurity at any point in their life, underscoring a prolonged food-secure environment.

Determinant Example: The medical report does not indicate any signs of the patient experiencing hunger or struggling with food accessibility or affordability, attesting to persistent food security.

Determinant Example: The patient's history does not mention any challenges related to food access or financial constraints preventing them from obtaining and enjoying meals without any difficulty, validating sustained food security.

Determinant Example: The patient states that they have never experienced food insecurity or gone without an adequate diet due to financial issues or scarcity of food, reinforcing their ongoing food security status.

Determinant Example: The medical report does not contain any remarks regarding the patient facing difficulties in obtaining or affording food, suggesting a reliable source of nutrition throughout their lifetime.

Determinant Example: The patient confirms never experiencing hunger or any issues related to obtaining or affording nutritious food, reflecting a consistent food security status.

Determinant Example: There are no indications or mentions of food insecurity in the patient's medical records, indicating a stable and secure food environment for the individual.

Determinant Example: The medical report shows no indications of the patient facing challenges in acquiring or affording nourishing meals, suggesting a consistently adequate diet.

Determinant Example: The patient's history does not reflect any instances of food scarcity or struggles with food affordability, indicating a consistent state of food security.

Determinant Example: The patient states that they have never faced food insecurity or struggled to maintain an adequate food supply, reflecting a secure food situation for the individual.

Determinant Example: The medical report does not mention any obstacles or hardships related to the patient's access to nutritious food, indicating an uninterrupted availability of nourishing meals.

Determinant Example: The patient expresses having a stable and reliable source of food without any disruptions or difficulties, underscoring consistent food security.

Determinant Example: There are no recorded concerns or incidents indicating the patient has experienced food insecurity, suggesting a continuously secure food environment.

Determinant Example: The medical report does not contain any remarks regarding the patient's food security or any difficulties in accessing nutrition, highlighting a reliable food supply.

Determinant Example: The patient confirms having consistent access to a diverse range of nutritious food without any disruptions or hesitations, pointing towards a maintained state of food security.

Determinant Example: There are no documented concerns or incidents suggesting the patient has faced food insecurity, reinforcing a long-standing and ongoing food security.

Table S6. ChatGPT-generated sentences for negating presence of food insecurity

Determinant Example: The patient denies experiencing any physical abuse or violence from their spouse or partner.
Determinant Example: There is no indication of domestic violence or any form of abuse in the patient's medical history.
Determinant Example: The patient states that their relationship is free from any violence or abusive behavior.
Determinant Example: The patient reports a stable and harmonious family life without any instances of domestic violence.
Determinant Example: During the assessment, the patient mentions a safe and supportive home environment, devoid of domestic violence.
Determinant Example: The patient's social history reveals a healthy and respectful relationship with their spouse, void of any abuse or violence.
Determinant Example: The patient denies any involvement with or exposure to domestic violence in their household.
Determinant Example: The patient asserts that their partner has always maintained an attitude of respect and kindness, ruling out domestic violence concerns.
Determinant Example: There is no evidence of physical or emotional harm inflicted by a family member or partner, suggesting no involvement in domestic violence.
Determinant Example: The patient discloses having a loving and peaceful family life without any instances of domestic violence.
Determinant Example: The patient's social support network is strong and free from any abusive relationships or domestic violence.
Determinant Example: As per the patient's statement, their home is a safe space where domestic violence is nonexistent.
Determinant Example: The patient confirms that their current living situation is not characterized by domestic violence or any form of abuse.
Determinant Example: The patient mentions being in a stable and nurturing relationship without any signs of domestic violence.
Determinant Example: The patient's spouse or partner does not exhibit any behavior that suggests involvement in domestic violence.
Determinant Example: The patient's social history reflects a healthy and positive marital relationship without any domestic violence concerns.
Determinant Example: The patient states that their living environment is free from domestic violence or any form of abuse.
Determinant Example: There is no evidence of physical or emotional harm caused by family members, ruling out domestic violence involvement.
Determinant Example: The patient highlights a supportive and loving relationship with their partner, ensuring the absence of domestic violence.
Determinant Example: The patient emphasizes the absence of domestic violence, indicating a peaceful and safe home environment.
Determinant Example: The patient's family dynamics do not indicate any form of abuse or domestic violence.
Determinant Example: The patient confirms that their partner has never displayed any violent or abusive tendencies, ruling out domestic violence concerns.
Determinant Example: The patient denies experiencing any hostile or aggressive behavior from their spouse or partner, ruling out domestic violence.
Determinant Example: The patient asserts that their home is a sanctuary, free from any domestic violence or abuse.
Determinant Example: The patient describes their relationship as respectful and peaceful, devoid of any signs of domestic violence.
Determinant Example: The patient states that they have never been subjected to domestic violence or any form of abuse throughout their life.
Determinant Example: The patient's social history reflects a supportive and nurturing environment, free from domestic violence.
Determinant Example: The patient mentions a healthy dating history, devoid of any instances of domestic violence or abuse.
Determinant Example: The patient's current living situation is characterized by mutual respect and absence of domestic violence.
Determinant Example: The patient's partner does not exhibit any behavior that suggests involvement in domestic violence or abuse.
Determinant Example: The patient's support system comprises individuals who promote healthy relationships, free from domestic violence.
Determinant Example: The patient denies experiencing or witnessing any form of domestic violence within their family or relationship.
Determinant Example: The patient affirms that their marital relationship is built on love and respect, excluding the possibility of domestic violence.

Determinant Example: The patient states that their spouse or partner treats them with kindness and understanding, eliminating domestic violence concerns.
Determinant Example: The patient's family history does not include any instances of domestic violence or abuse.
Determinant Example: The patient describes their relationship as a haven of safety and security, without any domestic violence or abuse.
Determinant Example: The patient asserts that their partner has never shown any signs of violence or abuse, excluding the possibility of domestic violence.
Determinant Example: The patient's social circle consists of individuals who prioritize healthy relationships, minimizing the risk of domestic violence exposure.
Determinant Example: The patient mentions having a remarkable support system, ensuring a safe and nurturing environment without any domestic violence.
Determinant Example: The patient denies any involvement in or exposure to domestic violence within their immediate or extended family.
Determinant Example: The patient affirms that their relationship is built on trust and open communication, negating the occurrence of domestic violence.
Determinant Example: The patient's close friends and family members are unaware of any incidents or signs of domestic violence, indicating a healthy relationship.
Determinant Example: The patient reports having never encountered or witnessed any form of domestic violence, suggesting a safe surrounding.
Determinant Example: The patient highlights the absence of violence or abuse in their family life, underscoring a lack of domestic violence.
Determinant Example: The patient does not express any concerns or suspicions regarding their partner's involvement in domestic violence.
Determinant Example: The patient describes their home as a peaceful and loving environment, free from any domestic violence.
Determinant Example: The patient states that their partner has always been supportive and considerate, excluding the presence of domestic violence in their relationship.
Determinant Example: The patient's social history reveals a nurturing and empathetic support system, minimizing the risk of domestic violence exposure.
Determinant Example: The patient denies any instances of domestic violence in their past or present relationship, indicating a safe and healthy environment.
Determinant Example: The patient emphasizes the absence of any abusive behavior in their family or relationship, ruling out domestic violence concerns.
Determinant Example: The patient's family members and close friends confirm the absence of any signs or incidents of domestic violence, ensuring a secure environment.
Determinant Example: The patient affirms that their partner has never exhibited any violent or abusive tendencies, negating the possibility of domestic violence.
Determinant Example: The patient's social circle comprises individuals who foster positive relationships, reducing the risk of domestic violence exposure.
Determinant Example: The patient's spouse or partner does not engage in any behavior that suggests involvement in domestic violence or abuse.
Determinant Example: The patient's support system consists of individuals who prioritize safety and equality in relationships, minimizing the likelihood of domestic violence.
Determinant Example: The patient denies any personal experiences or knowledge of domestic violence within their family or relationship.
Determinant Example: The patient confirms that their relationship is characterized by love and understanding, free from domestic violence.
Determinant Example: The patient denies any involvement in or knowledge of domestic violence within their immediate or extended family.
Determinant Example: The patient's social history includes supportive relationships that promote emotional well-being and exclude the risk of domestic violence exposure.
Determinant Example: The patient asserts that their partner has never displayed any aggressive or violent behavior, negating the presence of domestic violence.
Determinant Example: The patient's close friends and family members have not noticed any signs or incidents of domestic violence, affirming a healthy relationship.
Determinant Example: The patient reports no instances of physical harm or emotional abuse, ruling out any suspicion of domestic violence.

Determinant Example: The patient describes their home as a sanctuary, free from any form of domestic violence or abuse.

Table S7. ChatGPT-generated sentences for presence of domestic violence

Determinant Example 1: The patient presented with multiple injuries, including bruising and fractures, consistent with a history of physical abuse.
Determinant Example 2: The patient reported feeling fearful and anxious due to ongoing emotional abuse from their partner.
Determinant Example 3: The patient disclosed experiencing coercive control and isolation tactics by their spouse, indicating a potential domestic violence situation.
Determinant Example 4: Family members confirmed witnessing the patient's partner exhibit aggressive behavior and physical violence towards them.
Determinant Example 5: The patient's medical history revealed a pattern of injuries consistent with previous instances of domestic violence.
Determinant Example 6: The patient expressed concerns about their safety at home and revealed a history of intimate partner violence.
Determinant Example 7: The patient reported feeling trapped and powerless due to financial dependency on their abusive partner.
Determinant Example 8: The patient displayed signs of depression and anxiety, common mental health consequences of prolonged domestic abuse.
Determinant Example 9: The patient shared their struggle with their partner's use of coercive control, including monitoring their activities and limiting access to resources.
Determinant Example 10: Friends mentioned observing the patient's partner frequently yelling and belittling them in public, indicating emotional abuse.
Determinant Example 11: The patient's children displayed behavioral issues, commonly associated with exposure to domestic violence in the household.
Determinant Example 12: The patient presented with unexplained injuries and mentioned feeling frightened to disclose the true cause, suggesting a potential domestic violence situation.
Determinant Example 13: The patient expressed feelings of guilt and self-blame, often experienced by survivors of domestic violence.
Determinant Example 14: The patient's consistent avoidance of discussing their home life raised concerns about potential domestic violence.
Determinant Example 15: The patient described a cycle of abuse, wherein periods of violence alternated with apologies and promises of change from their partner.
Determinant Example 16: The patient's medical records documented a history of recurring injuries caused by their partner, indicative of ongoing intimate partner violence.
Determinant Example 17: The patient displayed symptoms of post-traumatic stress disorder, potentially linked to past experiences of domestic violence.
Determinant Example 18: The patient's social interactions appeared limited, hinting at potential isolation due to an abusive relationship.
Determinant Example 19: The patient reported incidents of sexual assault by their partner, indicating a multifaceted abusive dynamic.
Determinant Example 20: The patient revealed that their partner often withheld medications and medical care as a form of control and abuse.
Determinant Example 21: The patient mentioned feeling isolated and disconnected from friends and family, a common consequence of domestic violence.
Determinant Example 22: The patient expressed concerns about seeking help due to fear of retaliation and escalated violence from their partner.
Determinant Example 23: The patient's primary care physician suspected intimate partner violence based on the presence of injuries inconsistent with their reported activities.
Determinant Example 24: The patient disclosed a pattern of financial manipulation and withholding of resources by their partner, suggestive of economic abuse.
Determinant Example 25: The patient's partner's escalating aggression raised concerns about potential harm or even lethality.
Determinant Example 26: The patient described feeling controlled and constantly monitored by their partner, implying a situation of domestic violence.
Determinant Example 27: The patient relayed instances of their partner sabotaging their contraception methods, suggesting reproductive coercion.
Determinant Example 28: The patient expressed feelings of depression and hopelessness, which can be associated with ongoing abuse and lack of support.
Determinant Example 29: The patient's children displayed signs of witnessing violence, as they often drew violent scenes or engaged in aggressive play.

Determinant Example 30: The patient's medical records documented a lack of prenatal care, a red flag for potential intimate partner violence during pregnancy.
Determinant Example 31: The patient's explanation for injuries contradicted their severity, suggestive of potential minimization due to fear or shame related to domestic violence.
Determinant Example 32: The patient disclosed being coerced into substance abuse by their partner as a means of maintaining control.
Determinant Example 33: The patient's partner's controlling behavior extended to monitoring and limiting their access to healthcare services.
Determinant Example 34: The patient's disclosure of forced sexual encounters revealed a traumatic aspect of their domestic relationship.
Determinant Example 35: The patient's reluctance to engage in open conversation and their visible discomfort signaled a possible history of domestic violence.
Determinant Example 36: The patient reported constant threats and manipulation by their partner to maintain control over their actions and decisions.
Determinant Example 37: The patient's friends expressed concern about the increase in their partner's possessiveness and jealousy, indicating potential for escalating abuse.
Determinant Example 38: The patient disclosed feeling unsafe at home due to their partner's constant anger and explosive temper.
Determinant Example 39: The patient's prolonged absence from work due to injuries raised suspicions of intimate partner violence impacting their daily life.
Determinant Example 40: The patient neglected their own health due to the prioritization of their partner's needs and demands, a common consequence of domestic abuse.
Determinant Example 41: The patient's history of frequent emergency room visits aligned with the pattern of injuries consistent with domestic violence.
Determinant Example 42: The patient's partner's history of domestic violence arrests raised concerns about potential danger and ongoing abuse.
Determinant Example 43: The patient's explanation for their injuries changed multiple times during the examination, indicating potential deception to hide domestic violence.
Determinant Example 44: The patient expressed intense fear of their partner finding out about their healthcare visits, reflecting a climate of control and intimidation.
Determinant Example 45: The patient's medical records revealed a pattern of sexually transmitted infections, pointing towards potential sexual coercion by their partner.
Determinant Example 46: The patient's family members reported witnessing instances of physical violence, highlighting the presence of domestic abuse.
Determinant Example 47: The patient displayed self-harming behaviors, potentially a coping mechanism developed due to domestic violence trauma.
Determinant Example 48: The patient disclosed that their partner often restricted their access to transportation, limiting their ability to seek help or escape.
Determinant Example 49: The patient's explanation for their injuries conflicted with the severity and nature of the wounds, raising concerns about potential domestic violence.
Determinant Example 50: The patient shared their partner's attempts to manipulate them through threats of exposing personal or sensitive information.
Determinant Example 51: The patient consistently showed signs of hypervigilance, a common symptom experienced by survivors of domestic violence.
Determinant Example 52: The patient disclosed feeling humiliated and degraded by their partner's verbal insults and derogatory language.
Determinant Example 53: The patient's social network had significantly shrunk since entering the relationship, indicating potential isolation due to domestic violence.
Determinant Example 54: The patient exhibited signs of substance abuse as a coping mechanism to numb the distress caused by their abusive relationship.
Determinant Example 55: The patient's partner's strict control over their finances limited the patient's ability to seek medical attention or escape the abusive situation.
Determinant Example 56: The patient's children demonstrated symptoms of anxiety and withdrawal, possibly resulting from witnessing domestic violence in their household.
Determinant Example 57: The patient's partner's repeated threats to harm loved ones or pets created an environment of fear, control, and emotional abuse.
Determinant Example 58: The patient disclosed instances of their partner forcing them to perform sexual acts against their will, indicating sexual coercion and abuse.

Determinant Example 59: The patient's partner monopolized decision-making, leading to a lack of autonomy and control over their own healthcare choices.
Determinant Example 60: The patient exhibited signs of traumatic bonding, often seen in survivors of domestic violence who struggle to leave abusive relationships.
Determinant Example 61: The patient's injuries appeared consistent with patterns seen in strangulation cases, raising concerns about potential lethality.
Determinant Example 62: The patient's worsening mental health symptoms correlated with the duration of their abusive relationship, suggesting a connection between domestic violence and psychological well-being.
Determinant Example 63: The patient's partner displayed extreme possessiveness, insisting on constant monitoring of the patient's phone calls, texts, and whereabouts.
Determinant Example 64: The patient disclosed a previous attempt to leave the abusive relationship, only to be coerced into returning through threats and manipulation.
Determinant Example 65: The patient experienced frequent panic attacks and nightmares, often linked to the traumatic experiences endured during domestic violence.
Determinant Example 66: The patient's partner's controlling behavior extended to monitoring and limiting the patient's access to contraception methods, highlighting reproductive coercion tactics.
Determinant Example 67: The patient expressed feeling ashamed and embarrassed about their situation, commonly experienced by survivors of domestic violence.
Determinant Example 68: The patient's partner's interference with their medical appointments and treatment plan demonstrated a clear power dynamic indicative of intimate partner violence.
Determinant Example 69: The patient's partner exhibited patterns of isolation by discouraging the patient from maintaining contact with family, friends, and support networks.
Determinant Example 70: The patient's financial struggles resulted from their partner's financial control and abuse, making it difficult for the patient to seek help or leave the abusive relationship.
Determinant Example 71: The patient's fear of repercussions prevented them from disclosing the true extent of their injuries and abuse, highlighting the pervasive nature of domestic violence.
Determinant Example 72: The patient's partner's possessive behavior escalated to monitoring their social media activities and online interactions, limiting the patient's support system outside the relationship.
Determinant Example 73: The patient expressed feeling trapped in an abusive relationship due to threats of harm to themselves, loved ones, or pets if they were to leave.
Determinant Example 74: The patient experienced frequent migraines and gastrointestinal issues, physical manifestations of stress commonly observed in survivors of domestic violence.
Determinant Example 75: The patient displayed signs of learned helplessness, a psychological state often resulting from prolonged exposure to domestic abuse.
Determinant Example 76: The patient disclosed a lack of insurance or stable income due to their partner's controlling behavior, making it harder to access medical assistance and escape the abusive situation.
Determinant Example 77: The patient's partner's isolation tactics extended to limiting the patient's access to transportation or confining them within the home, further perpetuating the course of domestic violence.
Determinant Example 78: The patient reported a significant decline in self-esteem and loss of identity, consequences frequently associated with emotional abuse in domestic relationships.
Determinant Example 79: The patient shared their partner's manipulation tactics, such as gaslighting and blaming, undermining the patient's perception of reality and self-worth.
Determinant Example 80: The patient's partner's withholding of pain medications exacerbated the patient's physical suffering, reflecting a form of control and abuse within the relationship.
Determinant Example 81: The patient exhibited chronic stress symptoms, including sleep disturbances and weight fluctuation, consistent with the effects of ongoing domestic violence.
Determinant Example 82: The patient disclosed their partner's attempts to isolate them from healthcare professionals by accompanying them to appointments or speaking on their behalf.
Determinant Example 83: The patient's medical history revealed a pattern of sexually transmitted infections, suggesting potential forced sexual encounters within the context of domestic violence.
Determinant Example 84: The patient's partner's explosive anger and aggression created an environment of constant fear, affecting the patient's mental and emotional well-being.
Determinant Example 85: The patient's partner prevented them from accessing birth control methods, restricting the patient's autonomy and reproductive choices, thereby demonstrating reproductive coercion.
Determinant Example 86: The patient's reluctance to disclose information due to fear of being overheard or monitored raised concerns about their partner's control and intimidation tactics.
Determinant Example 87: The patient expressed difficulty concentrating and making decisions, common cognitive outcomes resulting from the prolonged stress of domestic abuse.

Determinant Example 88: The patient shared their partner's history of threatening suicide or self-harm as a means to manipulate and control the patient.
Determinant Example 89: The patient's anxiety symptoms intensified when discussing their partner, indicating the significant impact of the abusive relationship on the patient's mental health.
Determinant Example 90: The patient's partner's excessive jealousy and possessiveness impeded the patient's ability to engage in social activities or maintain relationships outside the abusive dynamic.
Determinant Example 91: The patient's injuries were consistent with defensive wounds, suggesting the patient had attempted to protect themselves during an episode of violence.
Determinant Example 92: The patient's partner's coercive control tactics extended to controlling the patient's access to essential medications, leading to potentially severe health consequences.
Determinant Example 93: The patient mentioned feeling isolated from their own cultural community due to their partner's influence and attempts to sever important connections.
Determinant Example 94: The patient shared their partner's threats to reveal private medical information as a way to maintain control and suppress the patient's autonomy.
Determinant Example 95: The patient's partner's economic abuse involved restricting the patient's access to financial resources, making it difficult for the patient to escape the abusive relationship.
Determinant Example 96: The patient displayed symptoms of dissociation and emotional numbing, psychological effects commonly experienced by survivors of prolonged domestic violence.
Determinant Example 97: The patient's partner's verbal abuse and insults targeted the patient's self-worth and body image, resulting in significant psychological distress.
Determinant Example 98: The patient disclosed that their partner frequently disregarded their consent during sexual encounters, pointing to a pattern of sexual coercion and violation.
Determinant Example 99: The patient's partner's aggression escalated when the patient attempted to seek support from friends or family, further isolating the patient from potential sources of help.
Determinant Example 100: The patient's fears of retribution and ongoing control by their partner hindered their willingness to seek assistance from healthcare professionals or report domestic violence incidents.

Table S8. ChatGPT-generated sentences for negating presence of domestic violence
